# Antibody responses in *Klebsiella pneumoniae* bloodstream infection: a prospective cohort study

**DOI:** 10.1016/j.lanmic.2024.100988

**Published:** 2025-02-11

**Authors:** Wontae Hwang, Paeton L Wantuch, Biana Bernshtein, Julia A Zhiteneva, Damien M Slater, Kian Hutt Vater, Sushmita Sridhar, Elizabeth Oliver, David J Roach, Sowmya R Rao, Sarah E Turbett, Cory J Knoot, Christian M Harding, Mohammed Nurul Amin, Alan S Cross, Regina C LaRocque, David A Rosen, Jason B Harris

**Affiliations:** Division of Infectious Diseases, Massachusetts General Hospital, Boston, MA, USA; Department of Pediatrics, Division of Infectious Diseases, Washington University School of Medicine, Saint Louis, MO, USA; Ragon Institute of Massachusetts General Hospital, Harvard and MIT, Cambridge, MA, USA; Ragon Institute of Massachusetts General Hospital, Harvard and MIT, Cambridge, MA, USA; Division of Infectious Diseases, Massachusetts General Hospital, Boston, MA, USA; Department of Pediatrics, Harvard Medical School, Boston, MA, USA; Division of Infectious Diseases, Massachusetts General Hospital, Boston, MA, USA; Division of Infectious Diseases, Massachusetts General Hospital, Boston, MA, USA; Division of Infectious Diseases, Massachusetts General Hospital, Boston, MA, USA; Division of Infectious Diseases, Massachusetts General Hospital, Boston, MA, USA; Department of Global Health, Boston University of Public Health, Boston, MA, USA; Division of Infectious Diseases, Massachusetts General Hospital, Boston, MA, USA; Department of Medicine, Harvard Medical School, Boston, MA, USA; Omniose, Saint Louis, MO, USA; Omniose, Saint Louis, MO, USA; Center for Vaccine Development and Global Health, University of Maryland School of Medicine, Baltimore, MD, USA; Center for Vaccine Development and Global Health, University of Maryland School of Medicine, Baltimore, MD, USA; Division of Infectious Diseases, Massachusetts General Hospital, Boston, MA, USA; Department of Medicine, Harvard Medical School, Boston, MA, USA; Department of Pediatrics, Division of Infectious Diseases, Washington University School of Medicine, Saint Louis, MO, USA; Division of Infectious Diseases, Massachusetts General Hospital, Boston, MA, USA; Department of Pediatrics, Harvard Medical School, Boston, MA, USA

## Abstract

**Background:**

*Klebsiella pneumoniae* is a leading cause of infection-related deaths globally, yet little is known about human antibody responses to invasive *K pneumoniae*. We sought to determine whether the O-specific polysaccharide antigen is immunogenic in humans with *K pneumoniae* bloodstream infection. We also sought to define the cross-reactivity of human antibody responses among structurally related *K pneumoniae* O-specific polysaccharide subtypes and to assess the effect of capsule production on O-specific polysaccharide-targeted antibody binding and function.

**Methods:**

In this prospective cohort study, we compared plasma antibody responses to O-specific polysaccharide in a cohort of consecutively enrolled patients with *K pneumoniae* bloodstream infection with controls, specifically a cohort of healthy individuals and a cohort of individuals with *Enterococcus* spp bloodstream infection. Patients were enrolled at the Massachusetts General Hospital, a tertiary hospital with affiliated clinics in the USA. We excluded patients whose isolates were not confirmed to be *K pneumoniae* by whole-genome sequencing. The primary outcome was the measurement of plasma IgG, IgM, and IgA antibody responses. We performed flow cytometry to measure the effects of *K pneumoniae* capsule production on O-specific polysaccharide antibody binding and O-specific polysaccharide antibody-mediated complement deposition, using patient isolates with variable levels of capsule production and isogenic capsule-deficient strains derived from these isolates.

**Findings:**

We enrolled 129 consecutive patients with suspected *K pneumoniae* bloodstream infection between July 24, 2021, and August 4, 2022, of whom 69 patients (44 [64%] male and 25 [36%] female) with confirmed *K pneumoniae* bloodstream infection were eligible for immunological evaluation. Common O-specific polysaccharide serotypes (O1, O2, O3, and O5) accounted for 57 (83%) of 69 infections. O-specific polysaccharide was immunogenic in patients with *K pneumoniae* bloodstream infection, and peak O-specific polysaccharide-IgG antibody responses in patients were ten-fold to 30-fold higher than antibody responses detected in healthy controls, depending on the serotype. There was cross-reactivity among similar O-specific polysaccharide subtypes, including the O1v1 and O1v2, O2v1 and O2v2, and O3 and O3b subtypes, as well as between the O1 and O2 types. Capsule produced by both hyperencapsulated and non-hyperencapsulated *K pneumoniae* inhibited O-specific polysaccharide-targeted antibody binding and function.

**Interpretation:**

O-specific polysaccharide was immunogenic in patients with *K pneumoniae* bloodstream infection, supporting its potential as a candidate vaccine antigen. The cross-reactivity observed between similar O-specific polysaccharide subtypes in patients with *K pneumoniae* bloodstream infection suggests that it might not be necessary to include all subtypes in an O-specific polysaccharide-based vaccine. However, these observations are tempered by the fact that capsule production, even in non-highly encapsulated strains, has the potential to interfere with O-specific polysaccharide antibody binding. This finding could limit the effectiveness of vaccines that exclusively target O-specific polysaccharide.

## Introduction

*Klebsiella pneumoniae* ranks as the fourth leading cause of infection-related mortality, responsible for approximately 790 000 deaths annually globally.^1^ *K pneumoniae* infections are caused by classical and hypervirulent pathotypes, distinguished by the ability of the hypervirulent type to cause invasive infections in healthy individuals. Hypervirulent *K pneumoniae* strains possess virulence factors, including siderophores and genes associated with capsule production.^2^ The dissemination of hypervirulent *K pneumoniae* that are also resistant to antibiotics poses a serious health threat.^3^ Despite the potential of vaccines against *K* pneumoniae,^4^ none are currently licensed or approved for use.

Given the need for *K pneumoniae* vaccines, there is an interest in identifying its antigenic repertoire.^5^ Capsular polysaccharide is one potential vaccine antigen since invasive *K pneumoniae* are often encapsulated. Additionally, high levels of capsule expression might mask other bacterial surface targets.^6^ However, not all invasive *K pneumoniae* express abundant capsule, and capsular polysaccharide production might be downregulated in vivo.^7,8^ The diversity of capsule also presents a challenge. Over 150 distinct *K pneumoniae* capsular polysaccharide structures are predicted,^9,10^ and a capsular polysaccharide-based vaccine would require more than 24 capsular antigens to reach 60% coverage of the currently circulating invasive *K pneumoniae* strains.^11^

The MrkA protein of *K pneumoniae*, a component of a type 3 fimbria, is another target for vaccine development. MrkA’s conservation across strains and role as an adhesin makes it an attractive target, and studies have shown that immunisation with MrkA can elicit strong IgG responses, reducing bacterial burden and providing protection in animal models of *K pneumoniae*, including murine models of sepsis and pneumonia.^12-14^

The O-specific polysaccharide, a component of lipopolysaccharide, is another target for *K pneumoniae* vaccine development.^9,11,15^ A 2020 survey estimated that population-based immunity to more than 80% of invasive *K pneumoniae* could be reached with a quadrivalent O-specific polysaccharide antigen vaccine targeting the O1, O2, O3, and O5 serotypes.^11^ However, there are gaps in our understanding of the role of O-specific polysaccharide antibody responses in immunity to *K pneumoniae*. Although O-specific polysaccharide is immunogenic in mice and rabbits,^16-18^ O-specific polysaccharide-targeted vaccines have variable efficacy in animal models, with protection dependent on the type of model (eg, the type of animal, the route of immunisation and infection, and the challenge strain).^6,19-22^ By contrast, human antibody responses to O-specific polysaccharide have not been characterised. Whether *K pneumoniae* O-specific polysaccharide is immunogenic in humans is relevant to vaccine development. Additionally, whether heterologous O-specific polysaccharide antigens induce cross-reactive antibody responses has implications for vaccine composition. Finally, it is unknown whether O-specific polysaccharide induces a functional antibody response in humans, and whether O-specific polysaccharide antibody binding is blocked by the variable levels of capsule produced by hypervirulent and classical *K pneumoniae*. To address these questions, we studied O-specific polysaccharide antibody responses in patients with *K pneumoniae* bloodstream infection. We measured the functional potential and cross-reactivity of O-specific polysaccharide antibody responses in humans. We also determined whether the capsule produced by invasive *K pneumoniae* isolates interfered with O-specific polysaccharide antibody binding and function.

## Methods

### Study design and participants

This prospective cohort study was done at Massachusetts General Hospital (Boston, MA, USA), a 1000-bed tertiary care hospital. We enrolled all identified patients, without age restrictions, with *K pneumoniae* bloodstream infection, as identified by the Massachusetts General Hospital clinical microbiology laboratory according to Clinical and Laboratory Standards Institute Guidelines, between July 24, 2021, and Aug 4, 2022. We excluded patients whose isolates were not confirmed to be *K pneumoniae* by whole-genome sequencing (WGS), as well as patients who had inadequate plasma collected due to death or insufficient follow-up. Follow-up was considered sufficient when the patient had at least one acute plasma sample collected 0–6 days after a positive *K pneumoniae* culture and at least one convalescent plasma sample collected 7–40 days after a positive *K pneumoniae* culture. Hypervirulent *K pneumoniae* were defined as strains which were *rmpA, iro,* and *iuc* positive.^23^ We compared immune responses in this *K pneumoniae* bloodstream infection cohort with a previously described prospectively enrolled cohort of consenting healthy adults (healthy controls) presenting for a routine outpatient consultation at Massachusetts General Hospital^24^ between July 2, 2019, and March 3, 2020, to establish baseline immune responses, and with a contemporaneously enrolled cohort of patients with *Enterococcus* spp bloodstream infection (*Enterococcus* bloodstream infection controls) admitted to Massachusetts General Hospital. The *Enterococcus* bloodstream infection controls were collected for this study due to their similar medical complexity to patients with *K pneumoniae* bloodstream infection, as measured by the Charlson Comorbidity Index.^25^ The inclusion criteria for the healthy controls were individuals aged 18 years or older attending the Massachusetts General Hospital Travel Clinic for routine pre-travel consultation. The inclusion and exclusion criteria for the *Enterococcus* bloodstream infection control cohort was the same as the *K pneumoniae* bloodstream infection cohort and they were enrolled in the same study protocol. Consent was waived from the *K pneumoniae* bloodstream infection or *Enterococcus* bloodstream infection control cohort, since only excess plasma samples collected for routine clinical use were required and no personally identifiable data were stored as part of the study. Consent was obtained from the healthy control cohort. The study was approved by the Mass General Brigham Institutional Review Board (study protocol 2021P001878 [*K pneumoniae* and bloodstream infection cohorts], and protocol 2019P001392 [health control cohort]; appendix 1 pp 35-37).

### Procedures and outcomes

Detailed procedures are included in appendix 1 (pp 3-10). Demographic (sex and age; data for gender and race or ethnicity were not systematically collected by the Massachusetts General Hospital) and clinical data, including data on immunosuppression (appendix 1 p 3), comorbidities, and other risk factors for invasive infection were extracted from the medical record, as described previously.^26^ Plasma was collected longitudinally at intervals determined on a case-by-case basis by practitioners for routine patient care over the course of each patient’s hospitalisation (until death or discharge) or on subsequent follow-up visits. Excess plasma was used for this study only after routine testing was completed.

The primary outcome of the study was the measurement of plasma IgG, IgM, and IgA antibody responses using a custom multiplexed bead assay to a panel of antigens, including exoprotein A of *Pseudomonas aeruginosa* (EPA) and human serum albumin (HSA) conjugated O-specific polysaccharide antigens (ie, O1v1, O1v2, O2v1, O2v2, O3/O3a , O3b, and O5), and the type 3 fimbrial protein, MrkA, for all three cohorts. We measured antibody responses to non-conjugated EPA and HSA carrier proteins and excluded plasma specimens that demonstrated reactivity with HSA or EPA from our analysis of O-specific polysaccharide responses due to potential binding to the carrier protein (appendix 1 p 11). We excluded individuals who were infected with *K pneumoniae* strains whose genotype did not encode a viable MrkA protein from an analysis of MrkA responses (appendix 1 p 12). A plasma dilution series, prepared from a mixture of patient serum, was used to normalise the median fluorescence intensity values between experiments. The secondary outcome was functional measurement of the immune response, including antibody-dependent complement deposition (ADCD) and antibody-dependent neutrophil phagocytosis (ADNP) targeting the O1v1 and O3b antigen (appendix 1 pp 5-6). We measured ADNP responses using flow cytometry to detect the phagocytosis of antigen-coupled beads by donor-derived granulocytes, and we measured ADCD responses using antigens conjugated with carboxylated beads, incubated with guinea pig complement, by flow cytometry as described previously.^27^ For a subset of patients, selected on the basis of variable levels of capsule expression by their infecting strains, we also measured the secondary outcome of plasma antibody binding and ADCD on whole bacterial cells using flow cytometry. This assessment was done using a pooled plasma sample or purified immunoglobulin from healthy controls, each patient’s paired plasma or purified immunoglobulin, *K pneumoniae* isolate, and an isogenic capsule-deficient mutant (Δ*wcaJ*), which were engineered to constitutively express green fluorescent protein. Plasma and purified immunoglobulin blocked with an excess of each specified antigen were used to determine O-specific polysaccharide-specific antibody binding and function (appendix 1 pp 8-9). Isogenic capsule-deficient mutants were constructed by deleting the *wcaJ* gene using either CRISPR-Cas9 and Lambda Red recombineering systems or the allelic exchange method (appendix 1 pp 7-8). Capsule expression for each strain and its isogenic Δ*wcaJ* mutant was measured using glucuronic acid quantification (appendix 1 pp 9-10).

### Statistical analysis

Summary statistics such as proportions, mean (SD), and median (IQR) were obtained for all variables. Separate bivariate analyses compared the distribution of characteristics between patients with *K pneumoniae* bloodstream infection and two distinct control groups: healthy controls and *Enterococcus* spp bloodstream infection controls. The significance of the difference in the distributions of the demographic characteristics was assessed using the *χ*^2^ test for categorical variables and the two-tailed *t*-test for continuous variables. Trends in antibody responses over time for patients with *K pneumoniae* bloodstream infection were visualised using locally weighted scatterplot smoothing (LOWESS) regression,^24^ with 95% CIs determined through 100 bootstrap resampling iterations. LOWESS, a non-parametric smoothing technique, facilitates the visual examination of the association of two variables over time. Differences in the distribution of IgG, IgM, and IgA antibody responses between groups (*K pneumoniae* bloodstream infection *vs* healthy controls; *K pneumoniae* bloodstream infection *vs Enterococcus* bloodstream infection controls), between different sources of infection in patients with *K pneumoniae* bloodstream infection, and between patients who were immunocompromised and immunocompetent with *K pneumoniae* bloodstream infection were evaluated using the Mann–Whitney U test, with 95% CIs of the medians determined through 10 000 bootstrap resampling iterations. This test was also applied to determine the statistical significance in the ADCD and ANDP comparisons. Additionally, Welch’s *t*-test was used to test the significance of the differences between groups, accounting for variability across technical replicates. Spearman rank correlations with 95% CIs, determined through 1000 bootstrap resampling iterations, were calculated to assess the relationship between capsular polysaccharide expression of the O1 *K pneumoniae* strains and the magnitude of the O1 antibody response, as well as between antibody levels across different *K pneumoniae* O-specific polysaccharide subtypes, using plasma from patients with *K pneumoniae* bloodstream infection, healthy controls, and *Enterococcus* bloodstream infection controls. All statistical analyses were conducted using Python (version 3.9.7) with the Scipy and Statsmodels packages, or GraphPad Prism 10. A two-sided p value of <0·05 was considered significant.

### Role of the funding source

The funder of the study had no role in study design, data collection, data analysis, data interpretation, or writing of the report.

## Results

**Figure 1:**
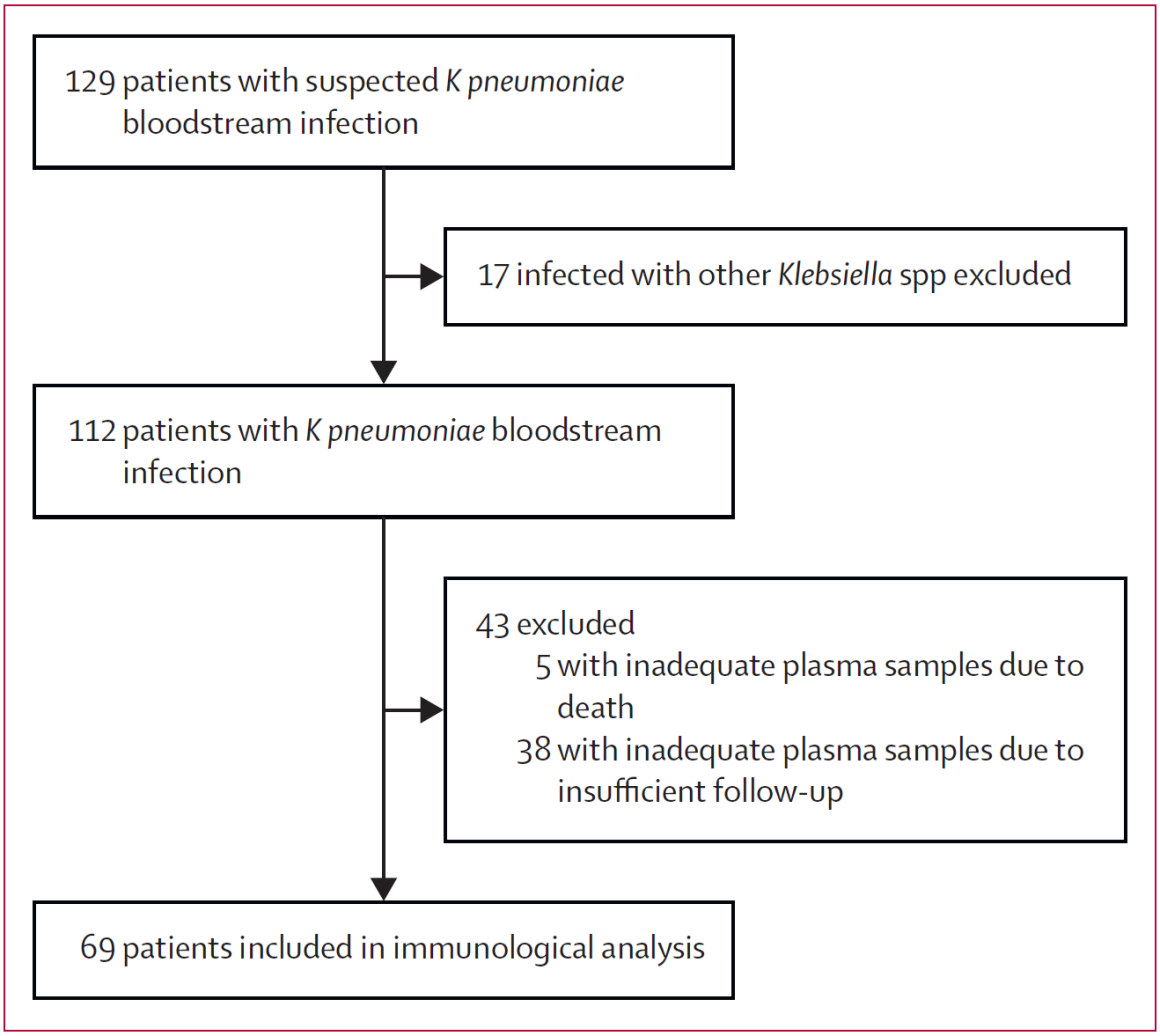
Study profile K pneumoniae=Klebsiella pneumoniae.

Between July 24, 2021, and Aug 4, 2022, we enrolled 129 patients with suspected *K pneumoniae* bloodstream infection over a 12-month study period (figure 1). The first 109 prospectively enrolled patients were also described in a previous study that evaluated the clinical and microbiological characteristics in a larger cohort of patients with *K pneumoniae* bloodstream infection.^26^ However, there was no description of their immune responses in the previous study.^26^ Of 129 patients, 17 were excluded because WGS identified *Klebsiella variicola* or *Klebsiella quasipneumoniae* rather than *K pneumoniae*, and 43 were excluded because of death or insufficient follow-up, leaving 69 eligible patients for measurement of O-specific polysaccharide antibody responses (figure 1). Among the 69 patients, three (4%) were infected with hypervirulent *K pneumoniae*. There were no significant differences in the age, sex, or immunocompromised status between the 69 patients who were included and the 43 patients with *K pneumoniae* bloodstream infection who were excluded from the immunological analysis (table). Age of patients ranged from 0 years to 92 years (median age 68 years [IQR 42–72]), and most were male (44 [64%] patients). The patients had a median Charlson Comorbidity Index of 5 (3–7) and 35 (51%) of 69 patients were immunocompromised, mostly due to chemotherapy-induced neutropenia. Our study also included 36 healthy controls (demographically distinct) and 25 *Enterococcus* bloodstream infection controls (table).

Previous studies have shown that >80% of global *K pneumoniae* infections are assigned to serotypes O1, O2, O3, or O5, which are considered potential O-specific polysaccharide vaccine serotypes.^9,11^ O1 and O2 antigens contain repeating galactan subunits and O3 and O5 contain mannose repeats (appendix 1 p 13). In our cohort of patients with *K pneumoniae* bloodstream infection, these four serotypes accounted for 57 (83%) of 69 *K pneumoniae* bloodstream infections (table). The most identified capsular polysaccharide serotype was K2, which is associated with hypervirulent *K pneumoniae*.^3,28^ However, no single capsular polysaccharide type predominated, and K2 was identified in only three patients (appendix 1 pp 28-30).

**Figure 2:**
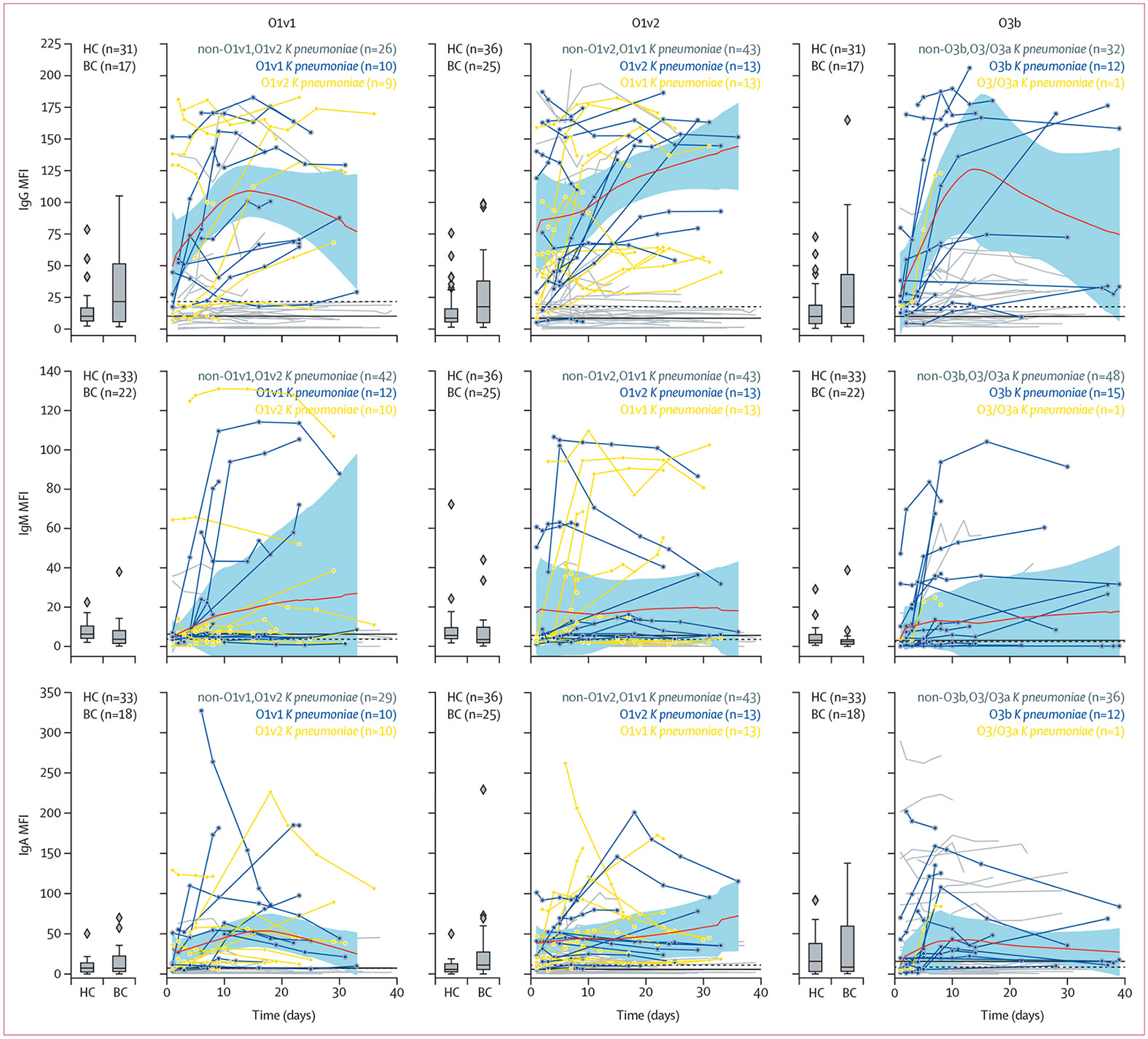
Longitudinal antibody responses to O1v1, O1v2, and O3b antigens in patients with *K pneumoniae* bloodstream infection The y-axis represents the MFI, divided by 1000. Boxplots show median MFI (IQR) from healthy controls and *Enterococcus* bloodstream infection controls. Longitudinal antibody responses in patients with *K pneumoniae* bloodstream infection are represented by individual lines, with each datapoint corresponding to a single sample at a given timepoint. The x-axis denotes the number of days since the first positive blood culture for *K pneumoniae* (day 0). Blue lines indicate antibody responses of patients with *K pneumoniae* bloodstream infection caused by infection with the homologous O-specific polysaccharide type, whereas orange lines represent patients with *K pneumoniae* bloodstream infection caused by heterologous O-specific polysaccharide subtype. Grey lines represent patients with *K pneumoniae* bloodstream infection caused by other O-specific polysaccharide serotypes. The red lines represent the locally weighted scatterplot smoothing regression applied to the blue lines, with 95% CIs shown as shaded blue areas. The solid black line represents the median of healthy controls, and the dashed black line represents the median of *Enterococcus* spp bloodstream infection controls. *BC=Enterococcus* bloodstream infection controls. HC=healthy controls. *K pneumoniae=Klebsiella pneumoniae*. MFI=median fluorescence intensity.

Patients with *K pneumoniae* bloodstream infection caused by O1v1, O1v2, and O3b strains, which were the most common in our cohort, exhibited increased IgG, IgM, and IgA antibody responses to their homologous O-specific polysaccharide serotype relative to both healthy controls and *Enterococcus* bloodstream infection controls. In aggregate, O-specific polysaccharide IgG responses increased over a 10-day period from the time of the initial *K pneumoniae*-positive blood culture (figure 2; appendix 1 pp 14-15).

**Figure 3:**
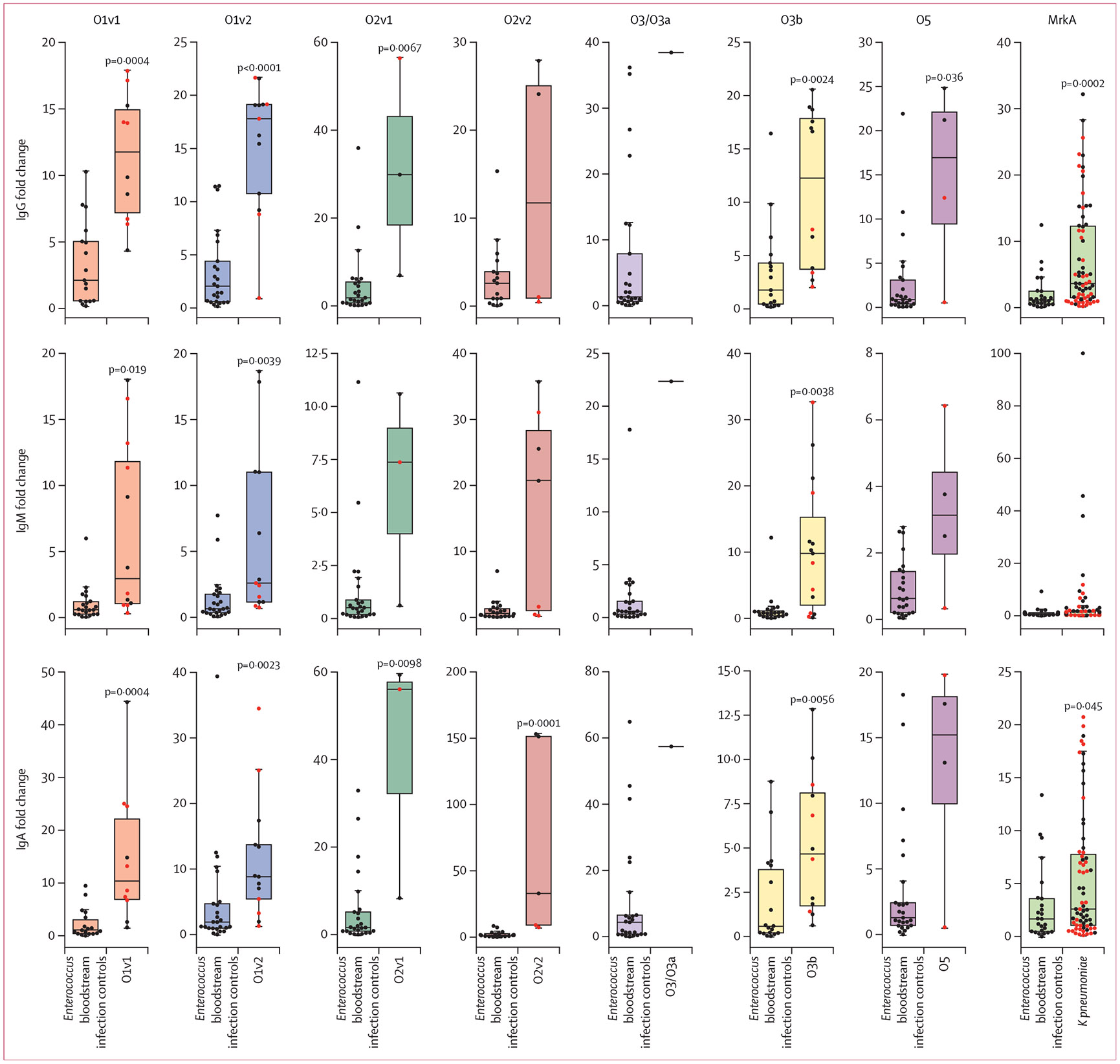
Homologous O-specific polysaccharide and MrkA antibody responses in *K pneumoniae* bloodstream infection Boxplots with IQRs depicting the highest antibody responses to homologous O-specific polysaccharide and MrkA in patients with *K pneumoniae* bloodstream infection, compared with *Enterococcus* bloodstream infection controls are shown. The y-axis represents the MFI divided by the median MFI of healthy controls. Red dots represent patients who were immunocompromised, whereas black dots represent patients who were immunocompetent. p values shown in the figure indicate the higher p values as determined by Mann–Whitney U test, comparing patients with *K pneumoniae* bloodstream infection to both healthy controls and *Enterococcus* bloodstream infection controls. The number of samples in each group is presented in appendix 1 (p 31). *K pneumoniae=Klebsiella pneumoniae*. MFI=median fluorescence intensity.

We compared the peak O-specific polysaccharide antibody responses in our cohort of patients with *K pneumoniae* bloodstream infection to those in healthy controls and *Enterococcus* bloodstream infection controls (figure 3). Patients infected with O1v1, O1v2, and O3b *K pneumoniae* demonstrated significant IgG, IgM, and IgA responses to their homologous O-specific polysaccharide compared with both groups of controls. Patients with bloodstream infection from the less common serotypes O2v1, O2v2, and O5 had significantly higher IgG or IgA responses than both sets of controls, but IgM responses were not significantly different from both sets of controls. Specifically, the median IgG response was 11·9-fold higher for O1v1 (95% CI 6·7–15·6; p<0·0001), 17·8-fold higher for O1v2 (10·8–19·1; p<0·0001), 12·0-fold higher for O3b (3·6–18·1; p< 0·0001), 29·9-fold higher for O2v1 (7·0–56·4; p=0·0009), and 16·8-fold higher for O5 (0·6–24·8; p=0·032) than the median IgG response in healthy controls. Patients with *K pneumoniae* bloodstream infection also had antibody responses to MrkA, with a 3·7-fold higher median IgG response (95% CI 2·8–5·3; p<0·0001) than the median IgG response in healthy controls. 24 (35%), five (7%), and 20 (29%) participants were excluded from the analysis of IgG, IgM, and IgA antibody responses to EPA-conjugated O-specific polysaccharide (O1v1, O2v2, and O3b), respectively, due to potential cross-reactivity with the EPA carrier protein (appendix 1 p 11). Additionally, two patients with *K pneumoniae* bloodstream infection were excluded from the analysis of the MrkA antibody response because of the absence of an intact MrkA protein in their infecting *K pneumoniae* strains (appendix 1 p 12). O-specific polysaccharide antibody responses did not significantly differ based on the patient’s source of infection (comparing a respiratory, urinary, or a gastrointestinal or hepatobiliary source; appendix 1 p 16).

We hypothesised that increased capsular polysaccharide expression might be associated with a lower antibody response to O-specific polysaccharide, so we measured the correlation between capsular polysaccharide expression and the magnitude of the O-specific polysaccharide antibody response. For this analysis, we focused on the O1 serotype since these were the most common. There was no significant correlation, either positive or negative, between the amount of capsular polysaccharide expressed by the infecting strain and the magnitude of the O1 IgG or IgA antibody response in patients with O1 *K pneumoniae* bloodstream infection. By contrast, a weak positive correlation was shown for O1v1 IgM responses (appendix 1 p 17).

Many individuals at risk for invasive *K pneumoniae* have compromised immunity, and our cohort included immunocompromised individuals. The conditions of patients who were immunocompromised, the possible sources of infection for each patient, and relative antibody responses to homologous O-specific polysaccharide and MrkA are presented in appendix 2. In our comparison of O-specific polysaccharide and MrkA antibody responses in patients with O1 *K pneumoniae* who were immunocompromised and non-immunocompromised, both groups exhibited IgG and IgA antibody responses to O1 antigens. By contrast, significant IgG responses to MrkA were observed predominantly among patients who were immunocompetent, although with exceptions that underscore the heterogeneity across the cohort. There were no significant IgM responses to O1 antigens shown in patients who were immunocompromised and to the MrkA antigen in patients who were immunocompetent (appendix 1 pp 18-19; appendix 2; appendix 3).

**Figure 4:**
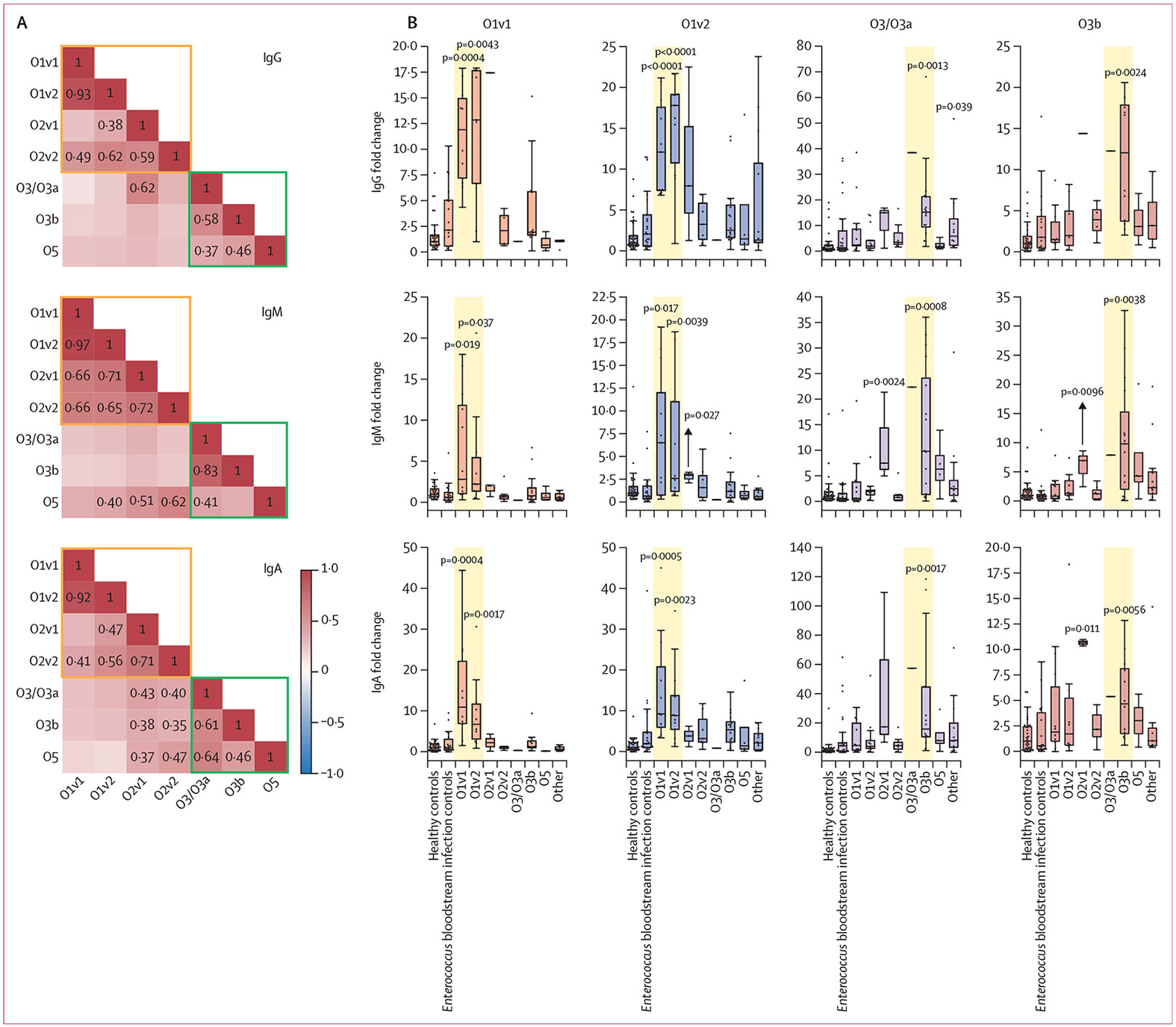
Cross-specific and cross-reactive antibody responses to closely related O-specific polysaccharide (A) A representative set of plasma samples with low cross-specificity to control antigens exoprotein A of *Pseudomonas aeruginosa* and human serum albumin (both MFI<200) were selected from patients with *K pneumoniae* bloodstream infection, healthy controls, and *Enterococcus* bloodstream infection controls. The heatmap shows the correlation between MFIs for different O-specific polysaccharide types, with numbers within the boxes indicating Spearman’s rank correlation values (*r*) with p<0·05; non-significant values are omitted. Galactan-based and mannan-based O-specific polysaccharide results are distinguished by orange and green lines, respectively. (B) Individual boxplots with IQRs display IgG, IgM, and IgA antibody responses for healthy controls, *Enterococcus* bloodstream infection controls, and the highest antibody responses from patients with *K pneumoniae* bloodstream infection in response to O1v1, O1v2, O3/O3a, and O3b antigens. The x-axis categorises plasma samples sourced from healthy controls, *Enterococcus* bloodstream infection controls, and patients infected with *K pneumoniae* with various O-specific polysaccharide types. The "Other" category includes O-specific polysaccharide types O4, O12, and unidentified O-specific polysaccharide. The y-axis represents the fold change in antibody responses relative to healthy controls, calculated as the MFI divided by the median MFI of healthy controls. Specifically, the results from patients infected with *K pneumoniae* strains that have homologous or heterologous O-specific polysaccharide subtypes compared with the antigen are highlighted with yellow backgrounds. p values shown in the figure indicate the higher p values as determined by Mann–Whitney U test, comparing *K pneumoniae* bloodstream infection with both healthy controls and *Enterococcus* bloodstream infection controls. Detailed information regarding the number of samples is available in appendix 1 (p 31). *K pneumoniae=Klebsiella pneumoniae*. MFI=median fluorescence intensity.

A cross-reactive response, which occurs when antigen stimulation generates antibodies that bind to structurally related antigens, can be immunologically advantageous. To assess O-specific polysaccharide antibody cross-reactivity, we measured the correlation in antibody responses between different *K pneumoniae* O-specific polysaccharide subtypes (figure 4A). IgG, IgM, and IgA antibody responses against O1v1 and O1v2 were all highly correlated in the study patients. Antibody responses were also correlated between the O2v1 and O2v2 subtypes, and the O3/O3a and O3b subtypes, as well as between O1 and O2 serotypes. In general, IgM responses were the most cross-reactive by this measure, although IgG and IgA responses also had significant cross-reactivity.

To measure cross-reactive immune responses to O-specific polysaccharide antigens, we measured antibody responses to heterologous O-specific polysaccharide antigens in patients with *K pneumoniae* bloodstream infection (figure 4B; appendix 1 pp 20-24). Patients with O1v1 or O1v2 *K pneumoniae* bloodstream infection had a robust response to both the homologous and heterologous O1 subtypes and O2 subtypes. Patients with O3b *K pneumoniae* bloodstream infection also had a robust response to the heterologous O3/O3a subtype. Patients with O2v2 *K pneumoniae* bloodstream infection also had significant IgG and IgA responses to the heterologous O2v1 antigen. Due to the small numbers of patients with O2v1, O3, and O5 infection, we were unable to assess heterologous antibody responses after exposure to these antigens.

To understand how O-specific polysaccharide-targeted antibodies might mediate immune function, we measured O-specific polysaccharide antibody-mediated ADNP and ADCD. Patients with O1 *K pneumoniae* bloodstream infection had ADNP and ADCD responses to the O1v1 antigen, whereas patients with O3b infection had ADNP and ADCD responses to the homologous O3b antigen (appendix 1 p 25). Additionally, ADNP responses appeared to exhibit some cross-reactivity between O1 and O3b; for example, patients with O1v2 infections showed a small but significant response to the O3b antigen.

Given that *K pneumoniae* bloodstream infection was shown to result in a functional O-specific polysaccharide antibody response, we assessed whether capsule production might interfere with O-specific polysaccharide antibody binding and ADCD responses. Four patients were selected based on variable levels of capsular polysaccharide expression in their infecting strain. This analysis included two patients infected with highly encapsulated *K pneumoniae* (patient 24 with hypermucoid classical *K pneumonia* and patient 128 with hypervirulent *K pneumonia*) and two patients infected with classical *K pneumonia* strains with low capsular polysaccharide expression (appendix 1 p 26).

**Figure 5:**
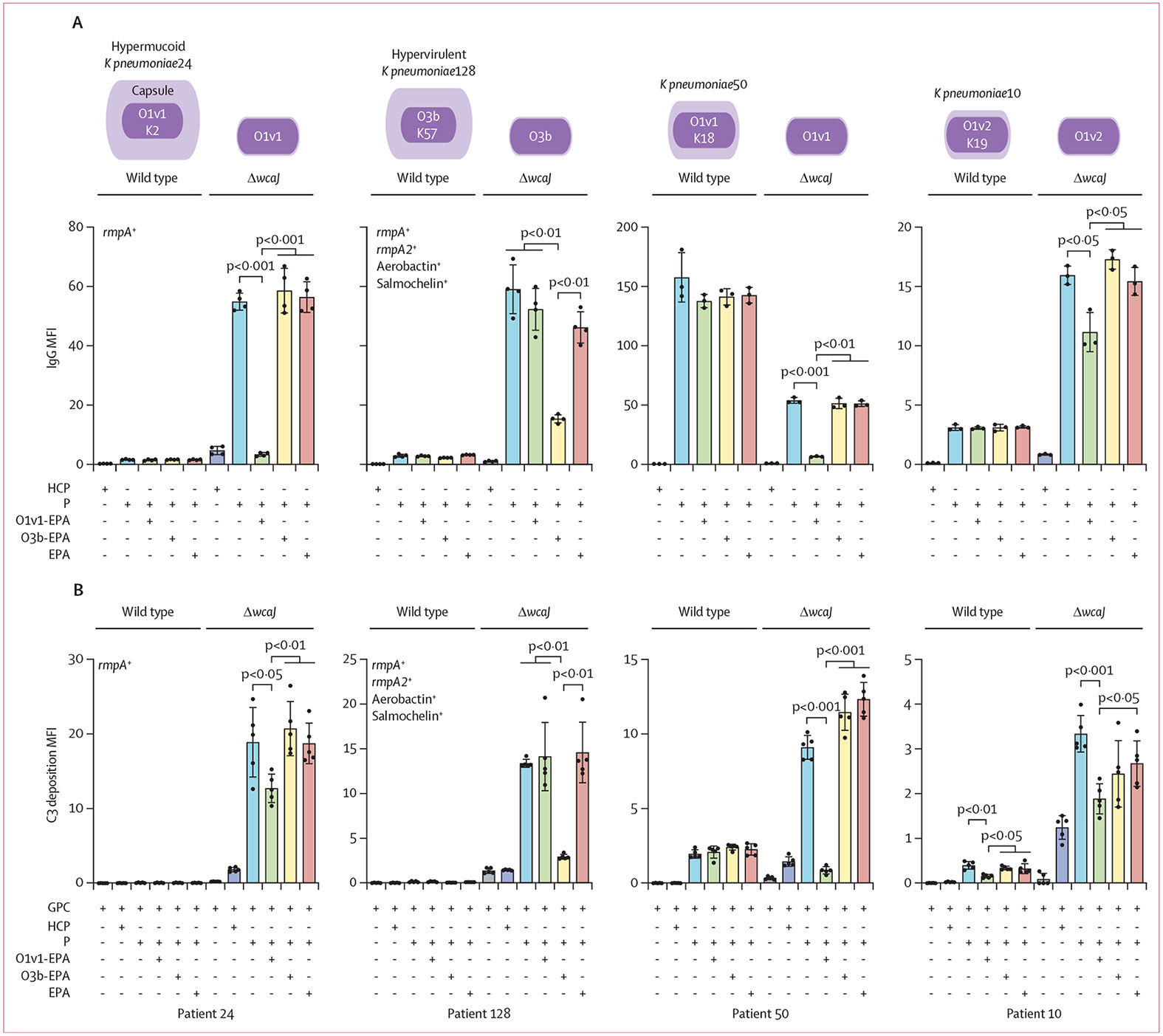
O-specific polysaccharide antibody binding to wild-type and capsule-deficient *K pneumoniae* and O-specific polysaccharide antibody-mediated complement deposition (A) A pooled plasma sample from 36 healthy controls was used as a baseline reference. Additionally, plasma samples with the highest antibody responses to the O-specific polysaccharide expressed by *K pneumoniae* isolates from patients with *K pneumoniae* bloodstream infection, and those adsorbed with O1v1–EPA, O3b–EPA, and EPA, were analysed. Specifically, these plasma samples and *K pneumoniae* isolates were obtained from patient 24, patient 128, patient 50, and patient 10. MFI values divided by 1000, representing the quantification of IgG binding to both wild-type *K pneumoniae* and its capsule-deficient mutant (Δ*wcaJ*), are displayed on the y-axis (n=4 for patients 24 and 128, n=3 for patients 50 and 10). The illustration above the charts represents the relative capsule expression levels in the four *K pneumoniae* wild-type strains and their Δ*wcaJ* mutants, based on data presented in appendix 1 (p 26). It also outlines the O-specific polysaccharide and capsular polysaccharide serotypes of *K pneumoniae* isolates as identified by Kleborate analysis in appendix 1 (pp 28-30), with specific characteristics of KPN24 and the hypervirulent KPN128 depicted in the figures. (B) Complement component C3 deposition on *K pneumoniae* strains facilitated by O-specific polysaccharide antibodies is shown. Purified immunoglobulins from the same plasma samples in (A), including HCP and P from patients with *K pneumoniae* bloodstream infection were used. Following opsonisation with both untreated and adsorbed immunoglobulins (with O1v1-EPA, O3b-EPA, and EPA), GPC was added. The subsequent C3 deposition on both wild type and Δ*wcaJ K pneumoniae* strains was measured and is presented as MFI divided by 1000, on the y-axis (n=5). EPA=exoprotein A of *Pseudomonas aeruginosa*. GPC=guinea pig complement. HCP=pooled healthy control plasma. HSA=human serum albumin. *K pneumoniae=Klebsiella pneumoniae*. MFI=median fluorescence intensity. P=plasma sample.

We measured the convalescent plasma IgG from each patient that bound to their infecting strain or to an isogenic capsular polysaccharide-deficient mutant (*ΔwcaJ*) derived from their infecting strain (figure 5A). Three of the four patients had an increase in IgG binding to their capsular polysaccharide-deficient mutants compared with the wild-type infecting strain.

To determine whether these antibody responses targeted O-specific polysaccharide, we pre-adsorbed the patients’ plasma using O-specific polysaccharide. A significant fraction of the non-capsular polysaccharide-targeted IgG antibody binding in all four of the patients was adsorbed by homologous O-specific polysaccharide (figure 5A). Similarly, we measured C3 complement deposition after the opsonisation of *K pneumoniae* strains and their corresponding capsular polysaccharide-deficient mutants from each patient. Notably, there was increased antibody-dependent C3 deposition on the capsular polysaccharide-deficient mutants compared with the wild-type infecting strain (figure 5B). In this experiment, pre-adsorption of immunoglobulin with O-specific polysaccharide of the serotype matching the target infecting strain resulted in a significant reduction in C3 deposition on capsular polysaccharide-deficient mutants (figure 5B).

## Discussion

There were three major findings in our study. First, O-specific polysaccharide was immunogenic in humans with *K pneumoniae* bloodstream infection, independent of capsule expression. Second, there was cross-reactivity among similar O-specific polysaccharide types in humans. Finally, even low levels of capsule expressed by classical *K pneumoniae* interfered with O-specific polysaccharide antibody binding and ADCD in vitro. These findings have implications for the design of *K pneumoniae* vaccines.

The finding that O-specific polysaccharide was a dominant antigen in human *K pneumoniae* infection highlights its potential as a vaccine component. Although this result is largely consistent with data from animal models,^16,17^ our study did not recapitulate findings that suggest the O2 serotype might be less immunogenic than other serotypes,^18^ and we found no evidence that the O2 serotype was less immunogenic in humans with *K pneumoniae* bloodstream infection.

An unexpected finding of our study was that O-specific polysaccharide was a dominant target of the antibody response, even in patients infected with heavily encapsulated *K pneumoniae* strains. This finding means that most individuals with *K pneumoniae* bloodstream infection had a response to O-specific polysaccharide, and a large fraction of antibodies that bound to the bacterial surface targeted O-specific polysaccharide. This observation contradicted our hypothesis that infection with strains with high capsular polysaccharide expression would be associated with reduced antibody responses to O-specific polysaccharide. The reason for this outcome might be because capsule production does not interfere with O-specific polysaccharide antigen presentation in the context of invasive disease (in which bacteria are destroyed and eliminated by immune cells), or because at certain stages of in-vivo infection capsular polysaccharide production might be downregulated, allowing for adequate presentation of O-specific polysaccharide antigen on the surface of live bacteria.^29,30^

Moreover, it was remarkable that serum antibodies in only one of four patients demonstrated higher antibody binding to the wild type than the corresponding capsular polysaccharide-deficient strain. This result underscores the relative antigenicity of O-specific polysaccharide versus capsular polysaccharide in *K pneumoniae* bloodstream infection, and suggests that a large fraction of the IgG response in patients with *K pneumoniae* bloodstream infection targets non-capsular polysaccharide outer membrane structures, but that these antibodies are blocked from binding by capsular polysaccharide produced by hyperencapsulated as well as non-hyperencapsulated strains.

Several explanations might account for this. One possibility is that capsule expression might be downregulated during invasive disease. However, this is unlikely given that animal models show capsule expression aids in pathogen survival during disseminated infection.^31^ Another hypothesis is that certain capsule types fail to induce a substantial antibody response and are therefore poorly immunogenic, allowing for immune evasion. This approach is supported by previous findings indicating that certain capsule types (eg, K1 and K2) can evade capture by macrophages, whereas others are more recognisable.^31^ A third hypothesis is that patients might be at risk for *K pneumoniae* bloodstream infection specifically because they are unable to mount an effective antibody response to the capsule.

Another finding of our study was that O-specific polysaccharide was immunogenic in an immunocompromised population with invasive *K pneumoniae*. 51% of patients in our cohort were immunocompromised, primarily due to chemotherapy or transplantation; nevertheless, IgG and IgA antibody responses to O-specific polysaccharide were relatively preserved in these patients, compared with responses to the protein antigen MrkA. This finding suggests that antibody class switching in this population might be induced by T-cell independent antibody responses in the presence of the innate immune response to *K pneumoniae* in the bloodstream.^32^ However, a previous study showed that T-cell dependent IgM antibody responses to bacterial capsules are necessary for full IgM production.^33^ Based on these findings, we hypothesise that suppressed T-cell activities in patients who are immunocompromised result in the hindrance of IgM antibody responses to O-specific polysaccharide. This observation might have implications for vaccine development, given that most individuals at risk for *K pneumoniae* have impaired immunity.^3,34^ Whether an O-specific polysaccharide-based vaccine, as opposed to natural *K pneumoniae* infection, could elicit antibody responses in an immunocompromised population would need to be evaluated empirically, as would the potential of a MrkA-based vaccine. Notably, although class switched O-specific polysaccharide antibody responses were relatively preserved in aggregate in this cohort, there was considerable individual variation in both O-specific polysaccharide and MrkA responses. To understand whether these antigens would serve as an effective vaccine in at-risk humans would require further study.

Our study highlighted IgG, IgM, and IgA cross-reactivity among related *K pneumoniae* serotypes, which has implications for vaccine development. Although the basis of cross-reactivity between O3 and O5 serotypes has been studied using monoclonal antibodies,^35,36^ a novel finding from our study is the observation of cross-reactivity between the O1v1 and O1v2 subtypes and between O2v1 and O2v2 subtypes, differing only by the addition of a single galactopyranose in the v2 genotypes, which underscores the potential for streamlined vaccine formulations.^16,37^ For example, the inclusion of both the O1v1 and O1v2 subtypes and both O3 and O3b subtypes in a multivalent *K pneumoniae* O-specific polysaccharide vaccine appears unnecessary. This efficiency in serotype selection could simplify vaccine design and improve its protective scope against common *K pneumoniae* serogroups.

Without established benchmarks of immunity against *K pneumoniae* infection in humans, we were unable to determine whether individuals mounted a protective response against re-infection. However, immunity against *K pneumoniae* is theorised to involve antibody binding to the bacterial cell, facilitating innate immune system functions, such as ADNP or ADCD, which are protective in animal models of *K pneumoniae* infection.^38,39^ Therefore, it is concerning that O-specific polysaccharide antibody-mediated complement deposition on the bacterial cell surface was blocked by capsular polysaccharide, not only in hyperencapsulated strains, but also by lower amounts of capsule produced by classical *K pneumoniae*. This finding underscores concern about the protective capacity of O-specific polysaccharide vaccines for invasive *K pneumoniae* infection.^6^

Our study has limitations. First, the loss of longitudinal data on immune responses in patients who died or were lost to follow-up introduces potential bias. Patients who died before the collection of follow-up plasma might have different immune responses to O-specific polysaccharide. Second, the investigation does not directly quantify antibody responses to specific capsular polysaccharide serotypes due to the non-dominance of any capsular polysaccharide serotype within our cohort; instead, it examines the influence of capsular polysaccharide by employing capsular polysaccharide-deficient strains. Third, the study was restricted to patients with blood culture-positive invasive *K pneumoniae* infection. Antibody responses during bloodstream infection might not be representative of the full spectrum of end-organ invasive *K pneumoniae* disease. However, we chose to focus on bloodstream infection, since it is a highly specific indicator of invasive infection, whereas mucosal surface cultures do not distinguish colonisation from invasive disease. Fourth, although our in-vitro experiments suggest that capsule production (even at low expression levels observed in some strains in non-highly encapsulated strains) interferes with O-specific polysaccharide antibody binding and complement deposition, this analysis was limited to a small subset of four patients, selected on the basis of the diversity of capsule expression, due to the low-throughput (albeit otherwise rigorous) nature of these experiments. Finally, it is worth noting that it is not possible to quantitate in-vivo expression of antigens including O-specific polysaccharide and MrkA during human infection, and that the O-specific polysaccharide and MrkA phenotype of infecting strains was inferred from their genotype. Thus, the variable or absent antigen expression of O-specific polysaccharide and MrkA could be a factor that limits antibody responses.^40^ Additionally, the expression of other antigens by co-infecting *K pneumoniae* strains might necessitate careful interpretation of cross-reactive immune responses.

In summary, preventing disease caused by *K pneumoniae* is crucial to the control of antimicrobial-resistant bacteria, a global health crisis. Our results demonstrate that O-specific polysaccharide is a dominant target of the immune response to invasive *K pneumoniae*, and that closely related O-specific polysaccharide subtypes are cross-reactive. However, the capsule might render O-specific polysaccharide-targeted antibodies incapable of protecting against infection. These findings have implications for vaccines targeting this formidable pathogen.

## Supplementary Material

mmc3

mmc2

mmc1

## Figures and Tables

**Table:** Demographic features of study participants and O-specific polysaccharide serotypes of infecting *K pneumoniae* strains

	Healthy controls (n=36)	*Enterococcus* bloodstream infection controls (n=25)	*K pneumoniae* group included in immunological analysis (n=69)	*K pneumoniae* group excluded from immunological analysis (n=43)	p value (included *K pneumoniae* group *vs* healthy controls)	p value (included *K pneumoniae* group *vs* bloodstream infection controls)	p value (included *K pneumoniae* group *vs* excluded *K pneumoniae* group)
Age					<0·001	0·88	0·97
Mean (SD)	47·8 (14·6)	63·6 (14·2)	63·1 (18)	62·9 (22·9)	··	··	··
Median (IQR)	47 (35–61)	66 (53–73)	68 (42–72)	65 (56–82)	··	··	··
Sex at birth					0·091	0·89	>0·99
Male	16 (44%)	17 (68%)	44 (64%)	27 (63%)	··	··	··
Female	20 (56%)	8 (32%)	25 (36%)	16 (37%)	··	··	··
Immunocompromised	NA	11 (44%)	35 (51%)	17 (40%)	NA	0·73	0·34
Charlson Comorbidity Score					NA	0·41	0·85
Mean (SD)	NA	4·72 (2·69)	5·25 (2·82)	5·38 (3·51)	··	··	··
Median (IQR)	NA	5 (3–7)	5 (3–7)	5 (2–8)	··	··	··
Missing	0	0	2 (3%)	3 (7%)	··	··	··
O-specific polysaccharide serotypes					NA	NA	NA
O1v1	··	··	13 (19%)	··	··	··	··
O1v2	··	··	13 (19%)	··	··	··	··
O2v1	··	··	3 (4%)	··	··	··	··
O2v2	··	··	7 (10%)	··	··	··	··
O3/O3a	··	··	1 (1%)	··	··	··	··
O3b	··	··	16 (23%)	··	··	··	··
O5	··	··	4 (6%)	··	··	··	··
Other*	··	··	12 (17%)	··	··	··	··

Among the 43 excluded patients, five (12%) died; however, among the 69 patients in immunological analysis there were eight (12%) deaths. Categorical variables were analysed using the *χ*^2^ test, whereas continuous variables were assessed using a two-tailed *t*-test. *O-specific polysaccharide serotypes of *K pneumoniae* isolates that were not O1, O2, O3, or O5. *K pneumoniae=Klebsiella pneumoniae*. NA=not applicable.

## Data Availability

All data and materials necessary to reproduce the findings of this study are comprehensively documented within the main paper and its appendices. The conditions of patients who were immunocompromised, the possible sources of infection for each patient, and relative antibody responses to homologous O-specific polysaccharide and MrkA are presented in appendix 2. p values, 95% CIs, and correlation values for antibody response comparisons are provided in appendix 3. All input datasets and analytical code are available online on GitHub.

## Research in context

### Evidence before this study

Despite the potential of O-specific polysaccharide as a vaccine antigen against *Klebsiella pneumoniae*, the immunogenicity of O-specific polysaccharide in humans remains largely unstudied, creating a knowledge gap with regard to vaccine development. We searched PubMed for publications from database inception to March 18, 2024, without language restrictions, using the terms “([Klebsiella pneumoniae] AND [O-specific polysaccharide]) AND (Antibody response)”. We found no studies addressing O-specific polysaccharide antibody responses in humans with *K pneumoniae* bloodstream infections. One study evaluated antibody response to a single lipopolysaccharide (which contains one subtype of O-specific polysaccharide) in humans with invasive *K pneumoniae* infection; however, in this study O-specific polysaccharide typing of the infecting strains and target antigen were not described.

### Added value of this study

Our investigation into O-specific polysaccharide immunogenicity in a human cohort marks an important advance. In our prospective cohort study, by analysing plasma antibody responses in 69 patients with *K pneumoniae* bloodstream infection, we found O-specific polysaccharide to be broadly immunogenic across all the types and subtypes examined, and there was cross-reactivity among structurally related O-specific polysaccharide antigens. We also showed that *K pneumoniae* capsule production inhibits O-specific polysaccharide antibody binding and the activation of complement on the bacterial surface, even in classical *K pneumoniae* strains expressing lower levels of capsule.

### Implications of all the available evidence

Although the immunogenicity and broad cross-reactivity of O-specific polysaccharide in individuals with *K pneumoniae* bloodstream infection suggests it is a promising vaccine candidate, the obstruction of O-specific polysaccharide antibody binding and engagement by the *K pneumoniae* capsule underscores the potential limitations of an exclusively O-specific polysaccharide-antigen based vaccine for *K pneumoniae*. Our study provides insights for the strategic development of vaccines aimed at combating infections with *K pneumoniae*, an important antimicrobial-resistant pathogen.

## References

[1] Collaborators GBDAR. Global mortality associated with 33 bacterial pathogens in 2019: a systematic analysis for the Global Burden of Disease Study 2019. Lancet 2022; 400: 2221–48. 36423648 10.1016/S0140-6736(22)02185-7 PMC9763654

[2] Russo TA, Olson R, Fang CT, Identification of biomarkers for differentiation of hypervirulent *Klebsiella pneumoniae* from classical *K pneumoniae*. J Clin Microbiol 2018; 56: e00776–18. 29925642 10.1128/JCM.00776-18 PMC6113484

[3] Choby JE, Howard-Anderson J, Weiss DS. Hypervirulent *Klebsiella pneumoniae*—clinical and molecular perspectives. J Intern Med 2020; 287: 283–300. 31677303 10.1111/joim.13007 PMC7057273

[4] Dangor Z, Benson N, Berkley JA, Vaccine value profile for *Klebsiella pneumoniae*. Vaccine 2024; 42: S125–41. 38503661 10.1016/j.vaccine.2024.02.072

[5] Assoni L, Girardello R, Converso TR, Darrieux M. Current stage in the development of *Klebsiella pneumoniae* vaccines. Infect Dis Ther 2021; 10: 2157–75. 34476772 10.1007/s40121-021-00533-4 PMC8412853

[6] Wantuch PL, Knoot CJ, Robinson LS, Capsular polysaccharide inhibits vaccine-induced O-antigen antibody binding and function across both classical and hypervirulent K2:O1 strains of *Klebsiella pneumoniae*. PLoS Pathog 2023; 19: e1011367. 37146068 10.1371/journal.ppat.1011367 PMC10191323

[7] Ernst CM, Braxton JR, Rodriguez-Osorio CA, Adaptive evolution of virulence and persistence in carbapenem-resistant *Klebsiella pneumoniae*. Nat Med 2020; 26: 705–11. 32284589 10.1038/s41591-020-0825-4 PMC9194776

[8] Walker KA, Miller VL. The intersection of capsule gene expression, hypermucoviscosity and hypervirulence in *Klebsiella pneumoniae*. Curr Opin Microbiol 2020; 54: 95–102. 32062153 10.1016/j.mib.2020.01.006 PMC8121214

[9] Follador R, Heinz E, Wyres KL, The diversity of *Klebsiella pneumoniae* surface polysaccharides. Microb Genom 2016; 2: e000073. 28348868 10.1099/mgen.0.000073 PMC5320592

[10] Wyres KL, Wick RR, Gorrie C, Identification of *Klebsiella* capsule synthesis loci from whole genome data. Microb Genom 2016; 2: e000102. 28348840 10.1099/mgen.0.000102 PMC5359410

[11] Choi M, Hegerle N, Nkeze J, The diversity of lipopolysaccharide (O) and capsular polysaccharide (K) antigens of invasive *Klebsiella pneumoniae* in a multi-country collection. Front Microbiol 2020; 11: 1249. 32595624 10.3389/fmicb.2020.01249 PMC7303279

[12] Choi M, Tennant SM, Simon R, Cross AS. Progress towards the development of *Klebsiella* vaccines. Expert Rev Vaccines 2019; 18: 681–91. 31250679 10.1080/14760584.2019.1635460 PMC6656602

[13] Lavender H, Jagnow JJ, Clegg S. *Klebsiella pneumoniae* type 3 fimbria-mediated immunity to infection in the murine model of respiratory disease. Int J Med Microbiol 2005; 295: 153–59. 16047414 10.1016/j.ijmm.2005.04.001

[14] Wang Q, Chang CS, Pennini M, Target-agnostic identification of functional monoclonal antibodies against *Klebsiella pneumoniae* multimeric MrkA fimbrial subunit. J Infect Dis 2016; 213: 1800–08. 26768253 10.1093/infdis/jiw021

[15] Kelly SD, Ovchinnikova OG, Müller F, Identification of a second glycoform of the clinically prevalent O1 antigen from *Klebsiella pneumoniae*. Proc Natl Acad Sci USA 2023; 120: e2301302120. 37428935 10.1073/pnas.2301302120 PMC10629545

[16] Wantuch PL, Knoot CJ, Robinson LS, A heptavalent O-antigen bioconjugate vaccine exhibits differential functional antibody responses against diverse *Klebsiella pneumoniae* isolates. J Infect Dis 2024; 230: 578–89. 38401891 10.1093/infdis/jiae097 PMC11420709

[17] Wagstaffe HR, Johnson M, Osman G, Martin P, Carranza P, Goldblatt D. The development of immunological assays to evaluate the level and function of antibodies induced by *Klebsiella pneumoniae* O-antigen vaccines. MSphere 2023; 8: e0068022. 36877023 10.1128/msphere.00680-22 PMC10117086

[18] Pennini ME, De Marco A, Pelletier M, Immune stealth-driven O2 serotype prevalence and potential for therapeutic antibodies against multidrug resistant *Klebsiella pneumoniae*. Nat Commun 2017; 8: 1991. 29222409 10.1038/s41467-017-02223-7 PMC5722860

[19] Clements A, Jenney AW, Farn JL, Targeting subcapsular antigens for prevention of *Klebsiella pneumoniae* infections. Vaccine 2008; 26: 5649–53. 18725260 10.1016/j.vaccine.2008.07.100

[20] Peng Z, Wu J, Wang K, Production of a promising biosynthetic self-assembled nanoconjugate vaccine against *Klebsiella pneumoniae* serotype O2 in a general *Escherichia Coli* host. Adv Sci (Weinh) 2021; 8: e2100549. 34032027 10.1002/advs.202100549 PMC8292882

[21] Hsieh PF, Lin TL, Yang FL, Lipopolysaccharide O1 antigen contributes to the virulence in *Klebsiella pneumoniae* causing pyogenic liver abscess. PLoS One 2012; 7: e33155. 22427976 10.1371/journal.pone.0033155 PMC3299736

[22] Liu Y, Pan C, Wang K, Preparation of a *Klebsiella pneumoniae* conjugate nanovaccine using glycol-engineered *Escherichia coli*. Microb Cell Fact 2023; 22: 95. 37149632 10.1186/s12934-023-02099-x PMC10163571

[23] Russo TA, Marr CM. Hypervirulent *Klebsiella pneumoniae*. Clin Microbiol Rev 2019; 32: e00001–19. 31092506 10.1128/CMR.00001-19 PMC6589860

[24] Iyer AS, Jones FK, Nodoushani A, Persistence and decay of human antibody responses to the receptor binding domain of SARS-CoV-2 spike protein in COVID-19 patients. Sci Immunol 2020; 5: eabe0367. 33033172 10.1126/sciimmunol.abe0367 PMC7857394

[25] Charlson ME, Pompei P, Ales KL, MacKenzie CR. A new method of classifying prognostic comorbidity in longitudinal studies: development and validation. J Chronic Dis 1987; 40: 373–83. 3558716 10.1016/0021-9681(87)90171-8

[26] Roach DJ, Sridhar S, Oliver E, Clinical and genomic characterization of a cohort of patients with *Klebsiella pneumoniae* bloodstream infection. Clin Infect Dis 2023. 10.1093/cid/ciad507 PMC10810715 37633257

[27] Bernshtein B, Ndungo E, Cizmeci D, Systems approach to define humoral correlates of immunity to *Shigella*. Cell Rep 2022; 40: 111216. 35977496 10.1016/j.celrep.2022.111216 PMC9396529

[28] Liu C, Guo J. Hypervirulent *Klebsiella pneumoniae* (hypermucoviscous and aerobactin positive) infection over 6 years in the elderly in China: antimicrobial resistance patterns, molecular epidemiology and risk factor. Ann Clin Microbiol Antimicrob 2019; 18: 4. 30665418 10.1186/s12941-018-0302-9 PMC6341648

[29] Cano V, March C, Insua JL, *Klebsiella pneumoniae* survives within macrophages by avoiding delivery to lysosomes. Cell Microbiol 2015; 17: 1537–60. 26045209 10.1111/cmi.12466

[30] Ares MA, Sansabas A, Rodríguez-Valverde D, The interaction of *Klebsiella pneumoniae* with lipid rafts-associated cholesterol increases macrophage-mediated phagocytosis due to down regulation of the capsule polysaccharide. Front Cell Infect Microbiol 2019; 9: 255. 31380298 10.3389/fcimb.2019.00255 PMC6650577

[31] Huang X, Li X, An H, Capsule type defines the capability of *Klebsiella pneumoniae* in evading Kupffer cell capture in the liver. PLoS Pathog 2022; 18: e1010693. 35914009 10.1371/journal.ppat.1010693 PMC9342791

[32] Allman D, Wilmore JR, Gaudette BT. The continuing story of T-cell independent antibodies. Immunol Rev 2019; 288: 128–35. 30874357 10.1111/imr.12754 PMC6653682

[33] Dolbec D, Lehoux M, de Beauville AA, Zahn A, Di Noia JM, Segura M. Unmutated but T cell dependent IgM antibodies targeting *Streptococcus suis* play an essential role in bacterial clearance. PLoS Pathog 2024; 20: e1011957. 38241393 10.1371/journal.ppat.1011957 PMC10829992

[34] Paczosa MK, Mecsas J. *Klebsiella pneumoniae*: going on the offense with a strong defense. Microbiol Mol Biol Rev 2016; 80: 629–61. 27307579 10.1128/MMBR.00078-15 PMC4981674

[35] Rollenske T, Szijarto V, Lukasiewicz J, Cross-specificity of protective human antibodies against *Klebsiella pneumoniae* LPS O-antigen. Nat Immunol 2018; 19: 617–24. 29760533 10.1038/s41590-018-0106-2

[36] Guachalla LM, Stojkovic K, Hartl K, Discovery of monoclonal antibodies cross-reactive to novel subserotypes of *K. pneumoniae* O3. Sci Rep 2017; 7: 6635. 28747785 10.1038/s41598-017-06682-2 PMC5529442

[37] Lam MMC, Wick RR, Judd LM, Holt KE, Wyres KL. Kaptive 2.0: updated capsule and lipopolysaccharide locus typing for the *Klebsiella pneumoniae* species complex. Microb Genom 2022; 8: 000800. 35311639 10.1099/mgen.0.000800 PMC9176290

[38] Banerjee K, Motley MP, Diago-Navarro E, Fries BC. Serum antibody responses against carbapenem-resistant *Klebsiella pneumoniae* in infected patients. MSphere 2021; 6: e01335–20. 33658281 10.1128/mSphere.01335-20 PMC8546725

[39] Kobayashi SD, Porter AR, Freedman B, Antibody-mediated killing of carbapenem-resistant ST258 *Klebsiella pneumoniae* by human neutrophils. MBio 2018; 9: e00297–18. 29535199 10.1128/mBio.00297-18 PMC5850326

[40] Hu F, Pan Y, Li H, Carbapenem-resistant *Klebsiella pneumoniae* capsular types, antibiotic resistance and virulence factors in China: a longitudinal, multi-centre study. Nat Microbiol 2024; 9: 814–29. 38424289 10.1038/s41564-024-01612-1 PMC10914598

