# Supplementary material for: Antibody responses in Klebsiella pneumoniae bloodstream infection: a prospective cohort study
Source: Lancet Microbe. Author manuscript; Available in PMC 2025 Jun 10. (PMC12150342; doi:10.1016/j.lanmic.2024.100988)
Supplement: mmc1 [file NIHMS2083509-supplement-mmc1.pdf]

THE LANCET
Microbe

Supplementary appendix 1

This appendix formed part of the original submission and has been peer reviewed. We post it as supplied by the authors.

Supplement to: Hwang W, Wantuch PL, Bernshtein B, et al. Antibody responses in *Klebsiella pneumoniae* bloodstream infection: a prospective cohort study. *Lancet Microbe* 2025. https://doi.org/10.1016/j.lanmic.2024.100988

Supplementary Materials for:

Antibody responses in *Klebsiella pneumoniae* bloodstream infection: a cohort study.

Authors: Wontae Hwang, Paeton L Wantuch, Biana Bernshtein, Julia A Zhiteneva, Damien M Slater, Kian Hutt Vater, Sushmita Sridhar, Elizabeth Oliver, David J Roach, Sowmya R Rao, Sarah E Turbett, Cory J Knoot, Christian M Harding, Mohammed Nurul Amin, Alan S Cross, Regina C LaRocque, David A Rosen, Jason B Harris

<u>Contents</u>:

Supplementary Methods (pages 3 to 10)

Figure S1 Longitudinal antibody responses to HSA and EPA, and exclusion strategy for EPA-positive patient samples (page 11)

Figure S2 MrkA homologues in 69 Kpn isolates (page 12)

Figure S3 The structures of galactan-based OPSs (O1, O2) and mannan-based OPSs (O3, O5) (page 13)

Figure S4 Longitudinal antibody responses to less common OPS subtypes in patients with Kpn BSI (pages 14 to 15)

Figure S5 Antibody responses in patients with Kpn infection from different sites (page 16)

Figure S6 Correlation between capsule amount of infecting strain and OPS antibody response (page 17)

Figure S7 Preserved OPS antibody responses in immunocompromised patients (pages 18 to 19)

Figure S8 Longitudinal antibody responses to homologous and heterologous OPS antigens in patients with Kpn BSI (pages 20 to 23)

Figure S9 Antibody responses to OPS and MrkA in patients with Kpn BSI (page 24)

Figure S10 OPS-specific ADNP and ADCD responses (page 25)

Figure S11 Capsule quantification of Kpn isolates and capsule knockout mutants (page 26)

Figure S12 Flow cytometry gating strategies (page 27)

Table S1 Kleborate results of 69 *Klebsiella pneumoniae* isolates (pages 28 to 30)

Table S2 The number of plasma samples used in Figure 3, Figure 4B, and Figure S9 (page 31)

Table S3 Primers used in this study (pages 32 to 33)

Supplementary References (page 34)

IRB Protocol Synopses (pages 35 to 37)

Materials and Methods

Enrollment of patients with *Klebsiella pneumoniae* blood stream infection and controls

This investigation was carried out at Massachusetts General Hospital (MGH), a 1000-bed tertiary care hospital which provides care for patients of all ages, with approval from the MassGeneral Brigham Institutional Review Board. We enrolled a cohort of all sequentially identified patients with *Klebsiella pneumoniae* (Kpn) blood stream infection (BSI), as identified by the clinical microbiology laboratory according to Clinical and Laboratory Standards Institute Guidelines, over a one-year period from 07/24/21 to 08/04/22. Demographic and clinical data were extracted from the medical record, as described previously.[1] Excess plasma used for this study was collected longitudinally for routine patient care over the course of each patients' hospitalization or on subsequent follow-up visits. We excluded patients whose isolates were not confirmed to be *Klebsiella pneumoniae* by whole genome sequencing. We excluded patients who had inadequate plasma collected due to death (5/43) or lack of follow-up visits (38/43) (Figure 1). We compared immune responses in the KPN BSI cohort with a previously described cohort healthy adults (HC) presenting for a routine outpatient pre-travel consultation at MGH,[2] and with a cohort of patients with *Enterococcus* spp. BSI (BC) hospitalized at MGH. One plasma sample per each healthy adult was collected. For patients with *Enterococcus* spp. BSI, a single plasma specimen between day 7 and day 35 following their first positive blood culture was collected. An immunocompromised patient was defined as having a history of cancer actively undergoing chemotherapy within the past 3 months, being a solid organ transplant recipient with ongoing immunosuppressive therapy, being on anti-inflammatory treatment, or having primary immunodeficiency (appendix 2). The study protocol was reviewed and approved by the Institutional Review Board (IRB) of Massachusetts General Hospital.

Whole genome sequencing (WGS) and analysis

We performed WGS on Kpn bloodstream isolates as described previously.[1] Libraries were prepared from genomic DNA isolated from a sub-culture of the first Kpn bloodstream isolate collected from patients. The sequencing was conducted by the Vantage core facility at Vanderbilt University Medical Center (Nashville, TN) using the Twist Biosciences NGS Kit. 150-base paired-end reads were generated on the Illumina NovaSeq6000 system. On average, each isolate produced 10 million reads. Adapter trimming was performed using Trimmomatic (v0.39)[3], and quality control was conducted with FastQC (v0.11.9).[4] Trimmed reads were assembled using SPAdes (v3.15.3).[5] Kleborate (v2.2.0)[6] was used to confirm Kpn identification and for *in silico* OPS and CPS serotyping. Hypervirulent Kpn

(hvKp) was classified based on the presence of either a complete *rmpA* or *rmpA2* gene in conjunction with complete aerobactin and salmochelin genes (Table S1).[7] BlastN analysis involved the comparison of *wbbY* reference sequence from the Kleborate database with the whole genome assemblies of O2v1 and O2v2 Kpn isolates. If fragmented *wbbY* genes were distributed across different contigs in these isolates, the OPS serotype was reclassified as O1v1 or O1v2. The Prokka tool (v1.13)[8] was utilized to identify open reading frames (ORFs) in the 69 whole genome assemblies of Kpn isolates. BlastP analysis of ORFs and the MrkA reference sequence (Figure S2) was performed, with results showing >90% identity and >85% gene length coverage considered as indicating a homologous protein of MrkA. MrkA homologous proteins were clustered using the CLUSTALW web-based tool[9] and visualized with Jalview[10] (Figure S2). In the comparison of MrkA homologues, patients infected with KPN6 and KPN55, lacking a MrkA homologue protein, were excluded from the analyses of the MrkA antibody response. For constructing *wcaJ* gene knockout mutants, WcaJ reference sequences of each CPS type (KPN10-K19, KPN24-K2, KPN50-K18, KPN128-K57) from the Kleborate database were used. BlastP searches with WcaJ reference sequences and ORFs of the four Kpn isolates were conducted, and the protein with >98% identity and >98% gene length coverage was identified as the WcaJ homologue. The Artemis tool[11] was employed to extract nucleotide sequences upstream and downstream of the *wcaJ* homologue for constructing a Δ*wcaJ* mutant.

Antigen preparation

O-antigens conjugated to Exoprotein A of *Pseudomonas aeruginosa* (EPA) (O1v1-EPA, O2v2-EPA, O3b-EPA), as well as EPA itself, were prepared as bioconjugates following established protocols as detailed elsewhere.[12] O-specific polysaccharides (OPS) conjugated to Human Serum Albumin (HSA) (O1v2-HSA, O2v1-HSA, O3/O3a-HSA, O5-HSA) were synthesized as follows: A mixture containing OPS, HSA, and sodium cyanoborohydride in a ratio of 3:1:6 (w/w/w) was stirred in 0.1 M borate buffer at pH 7.4 and maintained at 55°C for seven days, followed by dialysis in water. The MrkA protein (LSBio) served as the protein antigen for Kpn. EPA and HSA (Sigma-Aldrich) antigens were employed as controls for the bioconjugated antigens.

Multiplexed bead assay (MBA)

Magnetic beads, each corresponding to different bead regions, were conjugated with antigens following the protocol recommended by the xMAP® Antibody Coupling Kit (Diasorin). For the conjugation process, the amount of antigen used was 5µg for every 1 x 10^6 beads. Plasma from HC, BC and patients with Kpn BSI were diluted 1:100 in phosphate buffed saline (PBS) containing 1% BSA with 0.05% Tween20, and 5µl of the diluted plasma was added

to 384-Well Polystyrene Non-Binding Flat Bottom Microplate (Fisher Scientific Greiner Bio-One™) and incubated with the antigen coated bead mixture (840 beads per antigen) at room temperature for 2 hours, then washed three times. Subsequently, PE-conjugated mouse anti-human IgG, IgM, and goat anti-human IgA secondary antibodies (Southern Biotech) were added to each well and incubated for 1 hour. Following the incubation, the coated bead mixture was washed and resuspended in sheath fluid (Fisher Scientific). Fluorescence data from each well was acquired on a Luminex FlexMap3D. A standard dilution series, prepared from a mixture of serum from patients 7, 25, and 30, collected at 3, 44, and 7 days since the initial positive Kpn blood culture, was run independently on 4 experimental plates. Median Fluorescence Intensity (MFI) values were normalized across 4 individual MBAs by comparing standard curves. Specifically, the coefficient of variation (CV) of each antigen at each dilution factor between 4 standard curves was calculated. The 4 MFIs at the dilution factor with the lowest CV were used to normalize MFIs inter MBAs. In each antibody response (IgG, IgM, or IgA), patients with at least one plasma sample showing MFI values for the EPA antigen exceeding the 75th percentile (Q3) plus 1.5 times the interquartile range (IQR) of 36 healthy controls (calculated as Q3 + 1.5IQR) were identified as EPA-positive and excluded from the EPA-conjugated OPS (O1v1-EPA, O2v2-EPA, O3b-EPA) analyses (Figure S1). For example, patients with robust IgG responses to EPA were excluded from IgG analyses for O1v1, O2v2, and O3b antigens, while those with robust IgM responses to EPA were excluded from IgM analyses. In the cases of HSA-positive patients, MFIs to the HSA antigen are generally lower than Q3 + 1.5IQR MFI to the EPA antigen which is not expected to seriously affect MFIs to HSA-conjugated OPSs. Therefore, we did not exclude HSA-positive patients from the analyses of HSA-conjugated OPSs (Figure S1). In Figure 4A, representative plasma samples with the lowest antibody responses to the EPA antigen were selected from each patient with Kpn BSI. Plasma samples with MFIs below 200 for both HSA and EPA antigens, including those from healthy controls, bacteremic controls, and patients with Kpn BSI, were chosen. This approach was used to mitigate potential bias from varying antibody binding to different conjugative proteins. 29 (7 HC, 6 BC, and 16 patients with Kpn BSI), 30 (3 HC, 3 BC, and 24 patients with Kpn BSI), and 35 (9 HC, 4 BC, and 22 patients with Kpn BSI) human plasmas were retained for IgG, IgM, and IgA analyses, respectively. All Python modules used in the Luminex data analysis are presented in the analytical codes online on GitHub (https://github.com/WH551/Kpn-OPS-antibody-response). All results from the MBAs were visualized using seaborn (v0.11.2) and matplotlib (v3.4.3) in Python (v3.9.7).

Antibody-dependent neutrophil phagocytosis (ADNP)

Plasma collected the closest to day 10 after the first positive Kpn blood culture from patients with O1v1, O1v2, and O3b Kpn BSI was used to measure ADNP responses. Additionally, 36 plasma samples from healthy controls were included as a comparator group. O1v1 and O3b antigens were modified with DMTMM and coupled to carboxylated fluorescent beads (Thermo Fisher). To form immune complexes, each separately antigen-coupled bead was incubated for 2 hours at 37°C with diluted samples (1:200) and then washed to remove unbound immunoglobulins. The immune complexes were incubated for 1 hour with fresh blood neutrophils isolated from healthy donors using the EasySep Neutrophil Isolation Kit (StemCell). Following the incubation, cells were fixed with 4% paraformaldehyde and flow cytometry was performed to identify the percentage of cells that had phagocytosed beads as well as the number of beads that had been phagocytosed (phagocytosis score = % positive cells × Median Fluorescent Intensity of positive cells/10000). Flow cytometry was performed with an IQue (Intellicyt), and analysis was performed on IntelliCyt ForeCyt (v8.1) or using FlowJo V10.7.1.

Antibody dependent complement deposition (ADCD)

The same plasma samples used in the ADNP assay were utilized for ADCD. ADCD was conducted using a 384-well based customized multiplexed assay. O1v1 and O3b antigens were modified by 4-(4,6-dimethoxy[1,3,5]triazin-2-yl)-4-methyl-morpholinium and conjugated to Luminex Magplex carboxylated beads. To form immune complexes, a mixture of antigen-coupled beads was incubated for 2 hours at 37°C with diluted samples (1:200) and then washed to remove unbound immunoglobulins. Lyophilized guinea pig complement (Cedarlane) was resuspended according to manufacturer's instructions and diluted in gelatin veronal buffer with calcium and magnesium (Boston BioProducts). Resuspended guinea pig complement was added to immune complexes and incubated for 20 minutes at 37°C. Post incubation, C3 was detected with Fluorescein-Conjugated Goat IgG Fraction to Guinea Pig Complement C3 (Mpbio).

Bacterial strains and growth conditions

All Kpn isolates and *Escherichia coli* (*E. coli*) strains utilized in cloning experiments were cultured in Luria-Bertani (LB) medium with agitation at 200 rpm. Antibiotic-selective media, namely LB supplemented with apramycin (Apr), spectinomycin (Spec), and carbenicillin (Carbe), were used for genetic manipulation and bacterial growth carrying antibiotic-resistant plasmids. The antibiotic susceptibility profile for the four Kpn isolates (KPN10, KPN24, KPN50, and KPN128) included susceptibility to 30μg/ml Apr and 50~100μg/ml Spec, and *E. coli* S17-1 λpir was susceptible to 50μg/ml carbenicillin. Single bacterial colonies were selected from LB agar plates or LB agar

supplemented with antibiotics and inoculated into LB or LB with antibiotics for precultures, grown overnight at 30°C or 37°C with shaking at 200rpm. Precultures were then diluted 100-fold for subcultures and incubated similarly to the preculture conditions. All experiments involving Kpn were conducted under Biosafety Level 2 conditions.

Plasmid construction (pKPGFP, pDMS197-apr)

The plasmids pKPGFP and pDMS197-apr were engineered for this study. The backbone of pKPGFP was derived from pBad24-sfGFPx1 (addgene, #51558). In the pKPGFP plasmid, the *araC* gene was removed, and the *araBAD* promoter sequence was replaced with the *rpsL* promoter from pCasKP-apr (addgene, #117231) to facilitate endogenous GFP expression. The ampicillin-resistant gene (*ampR*) in pKPGFP was substituted with the apramycin-resistant gene from pCasKP-apr. pDMS197-apr utilized the pDMS197 plasmid backbone (addgene, #43831). In this plasmid, the tetracycline promoter and its corresponding resistance gene were replaced with the *ampR* promoter and apramycin-resistant gene from pKPGFP. Both plasmids were constructed using the Gibson assembly process. The primer design for the fragments for the two plasmids was facilitated through the NEBuilder Assembly Tool. These fragments were amplified with Q5 High-Fidelity DNA polymerase (NEB), and the linear PCR products were ligated using NEBuilder HiFi DNA assembly master mix (NEB). Subsequently, pKPGFP was transformed into DH5alpha chemically competent cells (Invitrogen), and pDMS197-apr was transformed into One Shot PIR1 chemically competent *E. coli* (Invitrogen). The transformed *E. coli* containing these plasmids were selected on LB agar with Apr.

***wcaJ* deletion**

To generate capsule-deficient mutants of three Kpn isolates with O1 serotypes (KPN10, KPN24, KPN50), we used the CRISPR-Cas9 and Red recombineering systems as described previously.[13] Briefly, the *wcaJ* homologue gene of the Kpn isolates was input into the CRISPRdirect website-based tool[14] to identify a suitable single guide RNA (sgRNA) sequence. The sequence-confirmed pSGKP-spec harboring sgRNA and homologue sequences for repairing sequence were co-transformed into electrocompetent Kpn isolates harboring pCasKP-apr. The repairing sequences included a synthetic 90-mer double-stranded DNA composed of each 45-mer of both upstream and downstream sequences of the *wcaJ* gene in constructing KPN50Δ*wcaJ* and approximately 1.3 kb homologous sequences composed of both ~0.65 kb upstream and ~0.65 kb downstream sequences of the *wcaJ* gene in constructing KPN10Δ*wcaJ* and KPN24Δ*wcaJ*. After incubating transformants on LB agar with Apr and Spec at

30°C, Sanger sequencing was performed to confirm sequence mismatches and identify Δ*wcaJ* mutants. Subsequently, pCasKP-apr and pSGKP-spec plasmids were cured.

Because this method failed to generate a *wcaJ* knockout mutant of KPN128, we used an alternative approach involving a two-step allelic exchange method using a modified pDMS197-apr plasmid. pDMS197-apr harboring flanking regions (~0.65 kb homologous sequences upstream and downstream of the *wcaJ* gene), pDMS197-apr_KPN128*wcaJ*, was electroporated into electrocompetent *E. coli* S17-1 λpir. Exponential-phase cultures of KPN128 and S17-1 λpir containing pDMS197-apr_KPN128*wcaJ* were spread on LB agar and LB agar with Apr overnight. Subsequently, bacterial mating was incubated for 5 hours on LB agar, followed by plating on LB agar with Apr and Carbe to identify chromosomally integrated pDMS197-apr_KPN128*wcaJ* in KPN128 (single-crossover events). Merodiploid colonies were streaked on no-salt LB supplemented with 15% sucrose at room temperature for over 24 hours to induce *sacB*-mediated plasmid removal (double-crossover events). The identification of the deletion mutant was achieved through colony PCR, and Sanger sequencing was performed to confirm the gene deletion in the KPN128.

Bacterial flow cytometry

Four Kpn isolates (KPN10, KPN24, KPN50, KPN128) and their capsule-deficient mutants (KPN10Δ*wcaJ*, KPN24Δ*wcaJ*, KPN50Δ*wcaJ*, KPN128Δ*wcaJ*), each harboring the pKPGFP plasmid, were obtained through electroporation and streaked on LB agar with Apr. The capsule-deficient mutants were used to assess antibody binding to capsule antigens by comparing antibody binding between wild-type strains expressing the capsule and their corresponding mutants lacking the capsule. Overnight cultures were grown in LB with Apr at 37°C, then washed with PBS containing 20% glycerol. Aliquots of cells were adjusted to a final OD of 1, and stored at -80°C for use in subsequent experiments. Plasmas exhibiting the highest antibody responses to the O1 antigens from patients with O1 Kpn BSI (patients 10, 24, and 50) and to the O3b antigen from patient with O3b Kpn BSI (Patient 128) were selected for this experiment. A pooled plasma sample consisting of 36 plasmas from healthy individuals served as a control plasma. Plasma complement was inactivated at 56°C for 30 minutes.

To neutralize specific antibodies, diluted plasma in flow buffer (2% BSA in PBS) was incubated with O1v1-EPA, O3b-EPA, and EPA antigens (final antigen concentration: 10μg/ml) at 37°C for 30 minutes with shaking at 200rpm. This pre-adsorbed plasma was used to assess the binding of O1v1 or O3b antibodies. Subsequently, 25μL of GFP-labeled bacteria and 25μL of diluted plasma or pre-adsorbed plasma were added to a V-

bottom 96-well plate. The final bacterial CFU in this reaction was approximately 1.5E+6, the final dilution factors of plasma were 1:400 for patient 24 and 1:1600 for patients 10, 50, and 128. After incubating at 37°C for 15 minutes with shaking at 200rpm, 200µL of flow buffer was added. The bacteria were then spun at 4,000g for 10 minutes at 4°C and washed. Subsequently, 50µL of 1:100 diluted goat anti-human IgG-PE secondary antibody (Southern Biotech) was added and resuspended by pipetting. After an incubation at room temperature, a second washing step with flow buffer was performed. The samples were then resuspended in flow buffer and the plate was read on a BD CSampler Plus (BD Biosciences) to collect at least 20,000 events. The threshold of FSC-H was set at 9,000, and fluidics speed was controlled not to exceed 2000/sec. Data was analyzed using FlowJo v10.9.0, and median MFIs of PE fluorescence were extracted after gating GFP-positive Kpn (Figure S12) (*N*=4 for patients 24 and 128, *N*=3 for patients 50 and 10).

C3 deposition on Kpn wild-type strains and their capsule-deficient mutants was evaluated utilizing Gelatin Veronal Buffer (GVB; Sigma). Immunoglobulins (Ig) from the previously mentioned plasma samples underwent were purified using NAb Protein A/G Spin Columns (Thermo Scientific). The purified Ig, at concentrations of 20µg/ml for samples from patients 10 and 128 and 10µg/ml for samples from patients 24 and 50, along with Ig pre-adsorbed with O1v1-EPA, O3b-EPA, and EPA antigens (final antigen concentration: 10µg/ml), were used for bacteria opsonization. Following this, 50µL of diluted guinea pig complement (Sigma) was incubated with the opsonized GFP-labeled bacteria at 37°C for 30 minutes with agitation at 200rpm. A subsequent washing step was performed using flow buffer before adding 50µL of 1:3200 dilution of goat anti-complement C3 polyclonal IgG (Invitrogen) and incubating at room temperature on a microplate shaker. After another washing step, 50µL of a 1:1000 dilution of rabbit anti-goat IgG conjugated with Alexa Fluor® 647 (abcam) was added to the samples, which were then incubated again. Following a final wash in flow buffer, flow cytometric analysis was conducted under the same conditions as the previous bacterial flow cytometry experiment. Median MFIs of APC fluorescence were extracted after gating GFP-positive Kpn (Figure S12) (*N*=5).

Capsule quantification

Glucuronic acid quantification was performed as described previously.[15] In brief, Kpn isolates and their Δ*wcaJ* mutants, cultured overnight, were concentrated and normalized to an OD600nm ranging from 3.00 to 3.50. After treatment with Zwittergent, the cultures underwent incubation at 50°C for 20 minutes, followed by centrifugation. The supernatant was mixed with cold ethanol, leading to capsule pellet formation upon centrifugation. After

resuspension in MilliQ water, a glucuronic acid standard dilution series was prepared. Sulfuric acid was added to each sample and standard, followed by incubation and cooling. Subsequently, 0.15% 3-hydroxydiphenol in 0.5% NaOH was introduced, and the solutions were spectrophotometrically measured at OD520nm. Capsule concentrations were determined by comparing sample OD measurements to the glucuronic acid standard curve (*N*=4).

Figure S1. Longitudinal antibody responses to HSA and EPA, and exclusion strategy for EPA-positive patient samples.

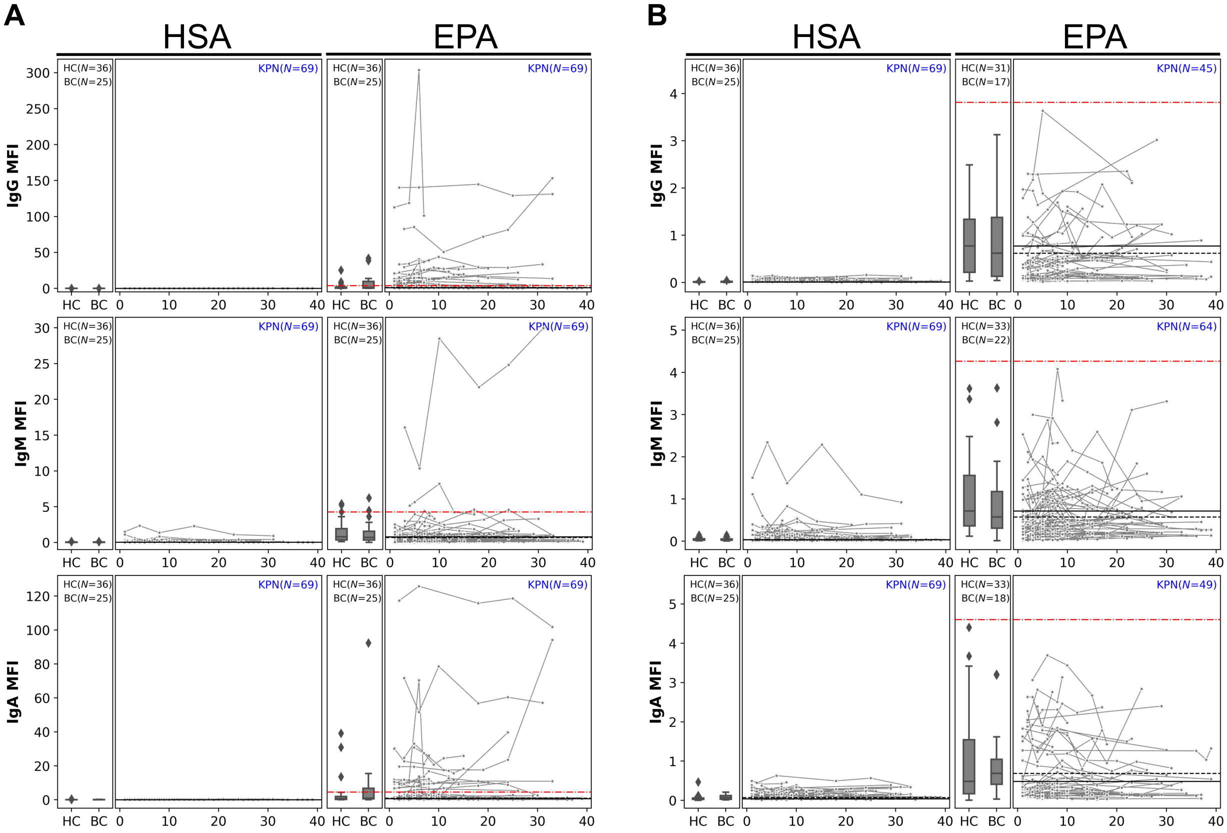

IgG, IgM, and IgA antibody responses to HSA and EPA antigens are presented. The Y-axis represents Median Fluorescence Intensity (MFI), divided by 1000. Boxplots compare MFIs to antigens in plasma samples from healthy controls (HC) and *Enterococcus* spp. bacteremic controls (BC). Longitudinal antibody responses in patients with Kpn BSI are shown by individual lines next to the boxplots, with each data point corresponding to a sample from a single patient at different time points. The X-axis denotes the date of plasma collection from patients with Kpn BSI after the first positive blood culture of Kpn (Day 0). Figures in **(A)** and HSA results in **(B)** utilized all patient plasma samples. EPA results in **(B)** used patient plasma samples after excluding samples from EPA-positive patients, which had at least one MFI higher than the cut-off MFI (Q3+1.5 IQR MFI to EPA antigen from 36 healthy controls, indicated by the red dot-dash line). The solid black line represents the median of HC, and the dashed black line represents that of BC. The number of patients included in each analysis is shown in each graph.

Figure S2. MrkA homologues in 69 Kpn isolates

>MrkA reference sequence
MKKVLLSAAMATAFFGMAAANAADTNVGGGQVNFFGKVTDVSCTVSVNGQGSDANVYLSPVTLTEVKAAAADTYLKPKSFTIDVSDCQAADGTKQDDVSKLGVN
WTGGNLLAGATAKQQGYLANTEAAGAQNIQLVLSTDNATALTNKIIPGDSTQPKAAGDASAVQDGARFTYYVGYATSTPTTVTTGVVNSYATYEITYQ

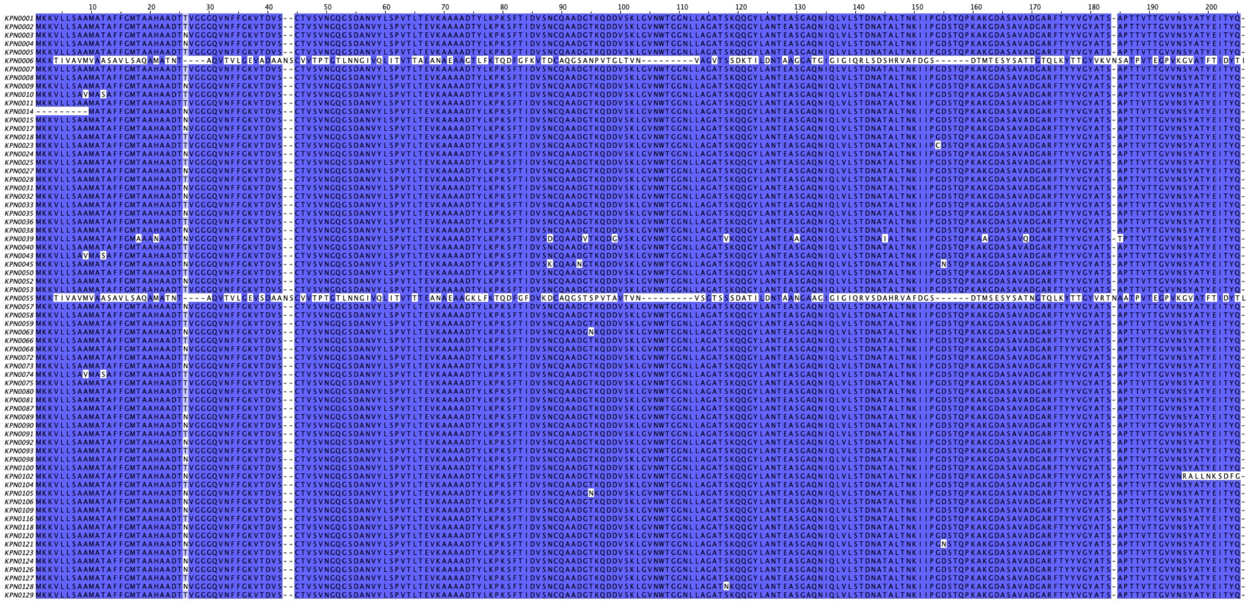

This illustrates the presence of MrkA homologues in 69 Kpn isolates. To identify these homologues, a Blastp analysis was performed using the MrkA reference sequence, in conjunction with the protein sequences obtained from the whole genomes of each Kpn isolate. The criteria for assigning a protein as a representative homologue included a similarity of over 90% and a gene length coverage of more than 85%. The identified MrkA homologues underwent clustering using the CLUSTAL web-based tool, and the resulting clusters were visualized using Jalview. In the visualization, dominant amino acid positions are highlighted in blue, aiding in the identification of conserved residues, while uncolored positions indicate rare occurrences.

Figure S3. The structures of galactan-based OPSs (O1, O2) and mannan-based OPSs (O3, O5)

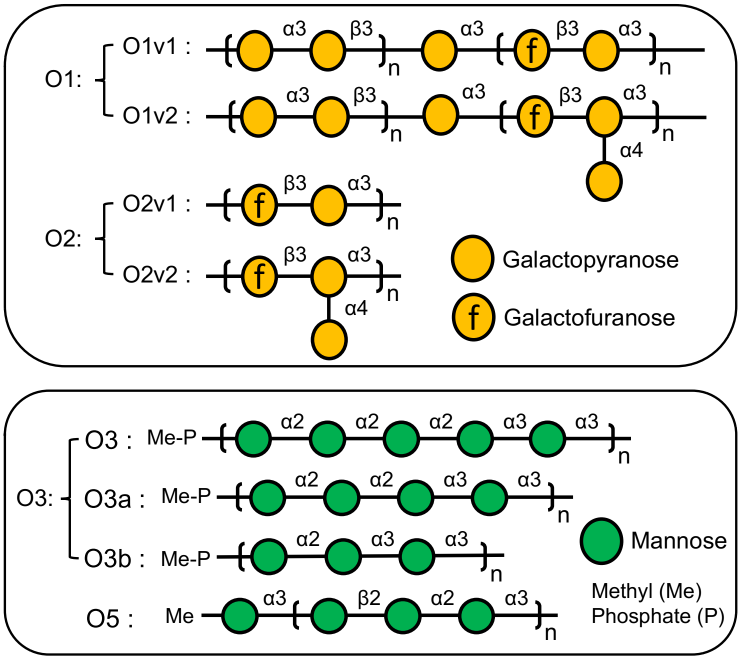

Figure S4. Longitudinal antibody responses to less common OPS subtypes in patients with Kpn BSI

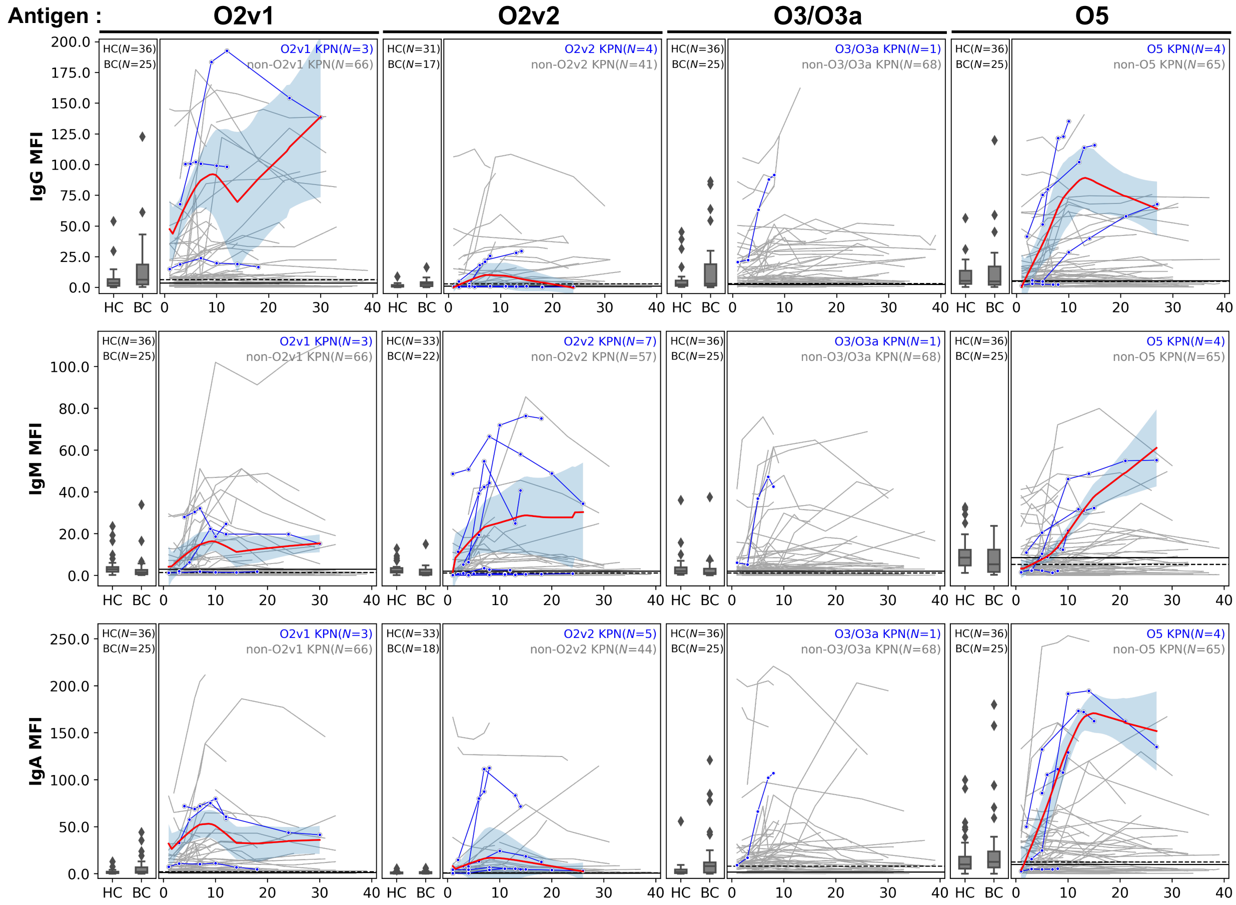

IgG, IgM, and IgA antibody responses to O2v1, O2v2, O3/O3a (O3), and O5 antigens over time were presented. The Y-axis represents Median Fluorescence Intensity (MFI), divided by 1000. Boxplots compare MFIs to antigens in plasma samples from healthy- (HC) and *Enterococcus* spp. bacteremic-controls (BC). Longitudinal antibody responses in patients with Kpn BSI are represented by individual lines next to boxplots, with each data point corresponding to samples from a single patient at different time points. X-axis denotes the date of plasma collection from patients with Kpn BSI after the first positive blood culture of Kpn (Day 0). Blue lines indicate antibody responses of patients bacteremic with Kpn isolates having the homologous OPS compared to the antigen, while gray lines represent patients bacteremic with Kpn isolates with heterologous OPS. Red lines illustrate the LOWESS regression applied to the blue lines, with 95% confidence intervals for LOWESS model shown as shaded blue areas. For O3/O3a Kpn infection, LOWESS regression analysis was not performed due to a small sample size. The solid

black line represents the median of HC, and the dashed black line represents that of BC. The number of patients included in each analysis is shown in each graph.

Figure S5. Antibody responses in patients with Kpn infection from different sites

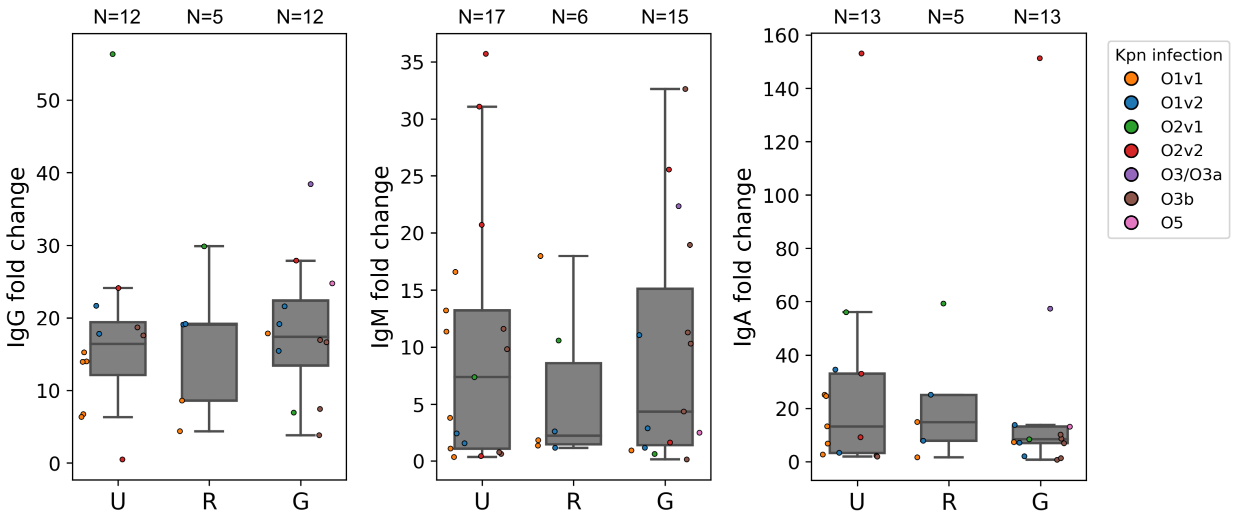

This figure compares the peak antibody responses to homologous OPS antigens in patients with Kpn BSI to the median MFI of healthy controls. Patients are categorized based on the source of infection: urinary (U), respiratory (R), or hepatobiliary and/or gastrointestinal (G). The analysis includes patients infected with Kpn strains exhibiting O1, O2, O3, and O5 OPS antigens (e.g., antibody responses to the O1v1 antigen in patients infected with the O1v1 Kpn strain, and antibody responses to the O2v1 antigen in patients infected with the O2v1 Kpn strain). Statistical differences between infection sites were determined using the Mann-Whitney U test.

Figure S6. Correlation between capsule amount of infecting strain and OPS antibody response

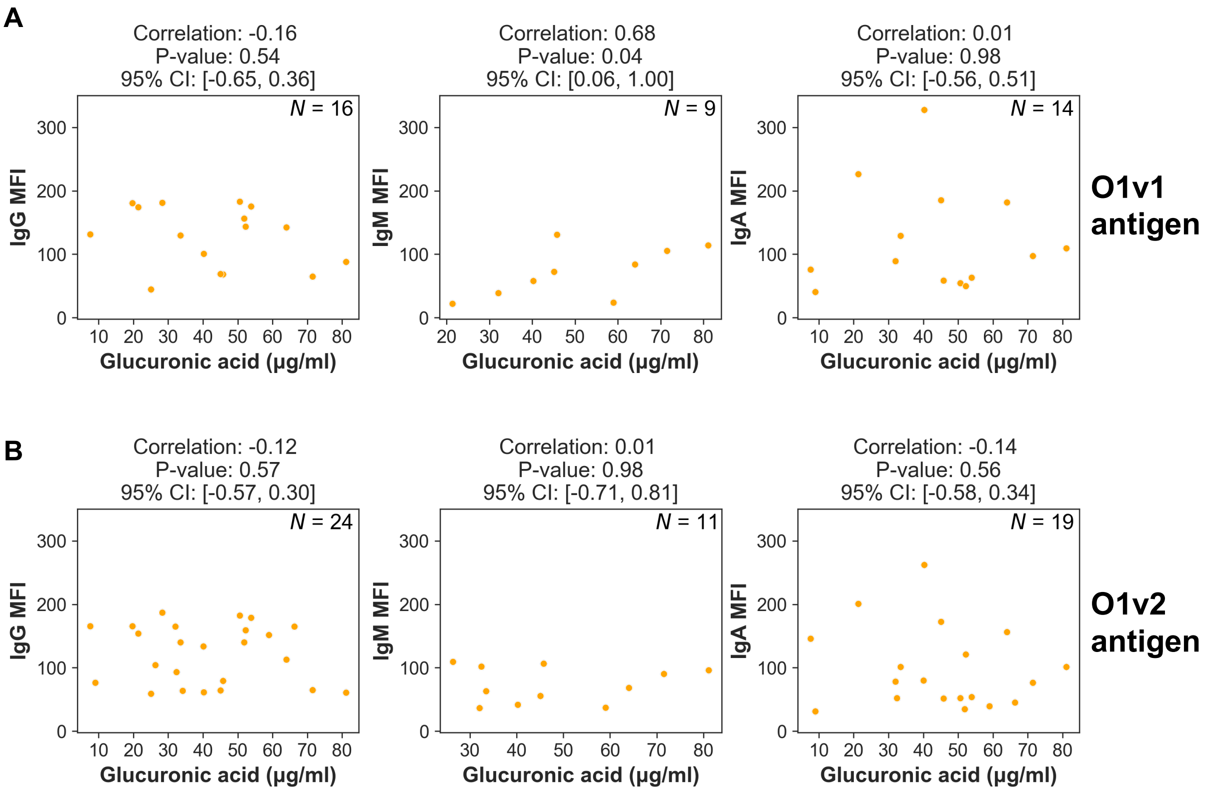

This figure illustrates the highest IgG, IgM, and IgA antibody responses (MFI divided by 1000) to O1v1 **(A)**, O1v2 **(B)** antigens in patients with O1 Kpn BSI, along with the glucuronic acid levels (indicative of capsule amount) in the associated O1 Kpn isolates. Plasma samples with MFI values below the 75th percentile (Q3) plus 1.5 times the interquartile range (IQR) of the healthy controls (Q3 + 1.5 IQR) were excluded as non-responsive samples. The figure provides the Spearman rank correlation values, P values, and 95% confidence intervals determined via bootstrap resampling, along with the number of samples used in the analysis.

Figure S7. Preserved OPS antibody responses in immunocompromised patients

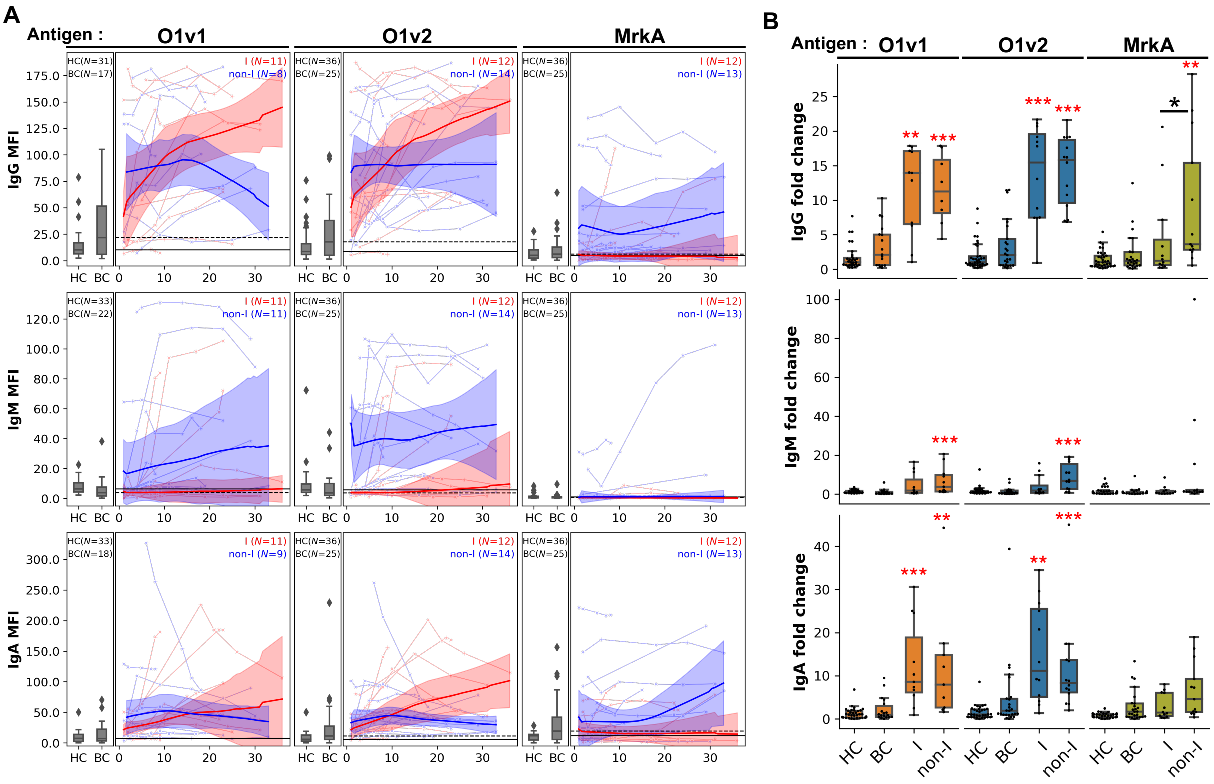

(A) O1v1, O1v2 and MrkA antibody responses in plasma samples from healthy controls (HC), patients with *Enterococcus* spp. BSI (BC), and patients with O1 Kpn BSI, were measured. The Y-axis presents MFI values, divided by 1000, and each boxplot displays antibody responses to antigens from HC and BC, while longitudinal antibody responses in patients with O1 Kpn BSI are depicted by individual lines next to boxplots. Each data point on a line corresponds to samples from a single patient at various time points. The X-axis represents the date when plasma was collected post the first positive blood culture for Kpn (Day 0). Results from patients with O1 Kpn BSI were categorized into immunocompromised patients (I) and non-immunocompromised patients (non-I). Red lines indicate antibody responses from I, blue lines represent antibody responses from non-I. Darker lines show the result of LOWESS regression applied separately to the I and non-I individuals, highlighting the trend in antibody responses over time, with 95% confidence intervals for LOWESS model shown as shaded areas. The solid black line represents the median MFI of HC, and the dashed black line represents that of BC. The number of patients for each analysis is shown in each graph. **(B)** The peak antibody responses to antigens from patients with O1 Kpn BSI are shown in fold change units relative to the median MFI of HC. Results are categorized into antibody responses from I

and non-I groups. Red asterisks indicate the higher P values when compared to both HC and BC groups using the Mann-Whitney U test. A black asterisk denotes the comparison of fold change between the I and non-I groups, also determined by the Mann-Whitney U test. * $0.01 \le P < 0.05$, ** $0.001 \le P < 0.01$, *** $P < 0.001$. The number of samples is the same as those in (A).

Figure S8. Longitudinal antibody responses to homologous and heterologous OPS antigens in patients with Kpn BSI

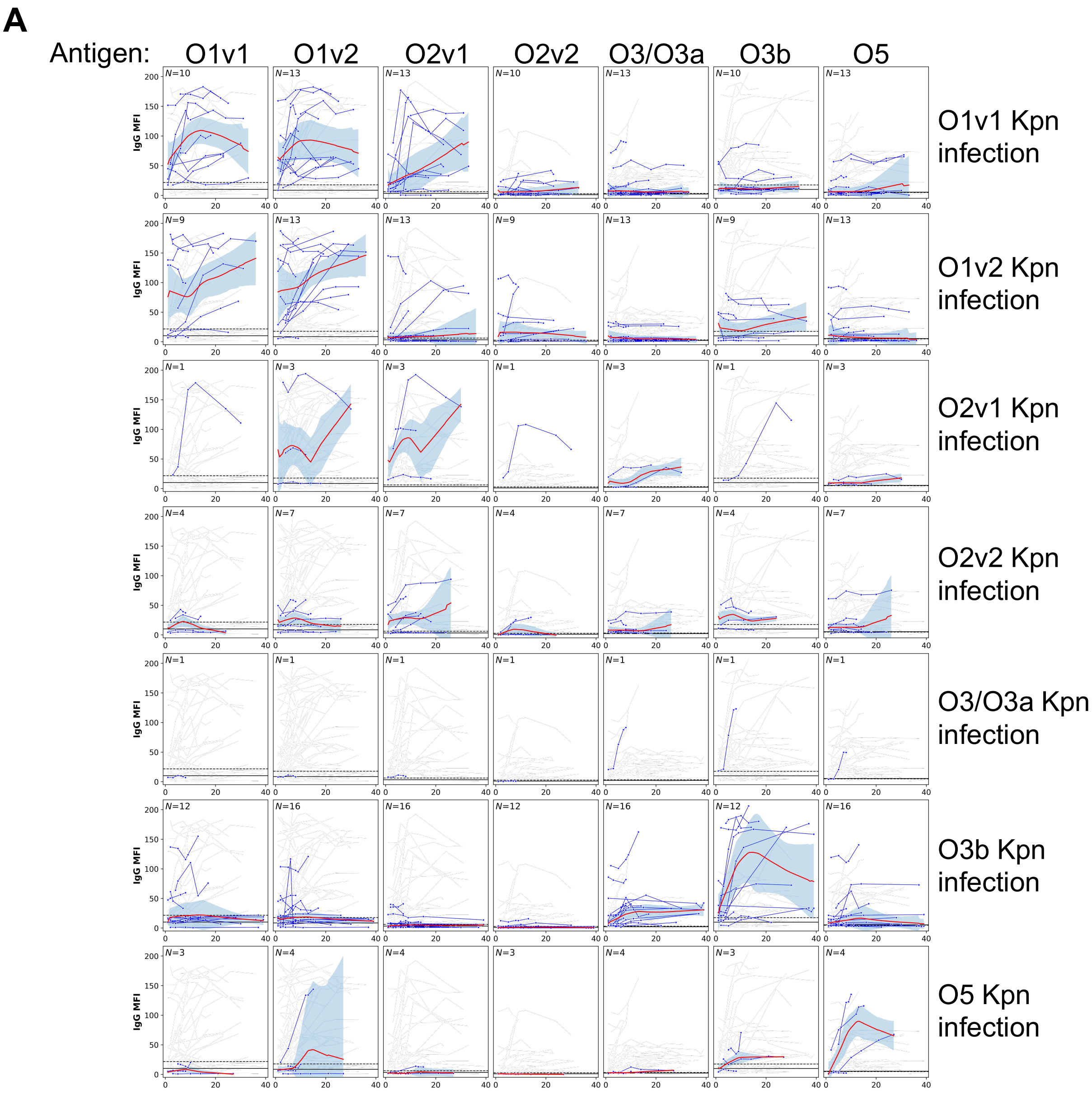

314

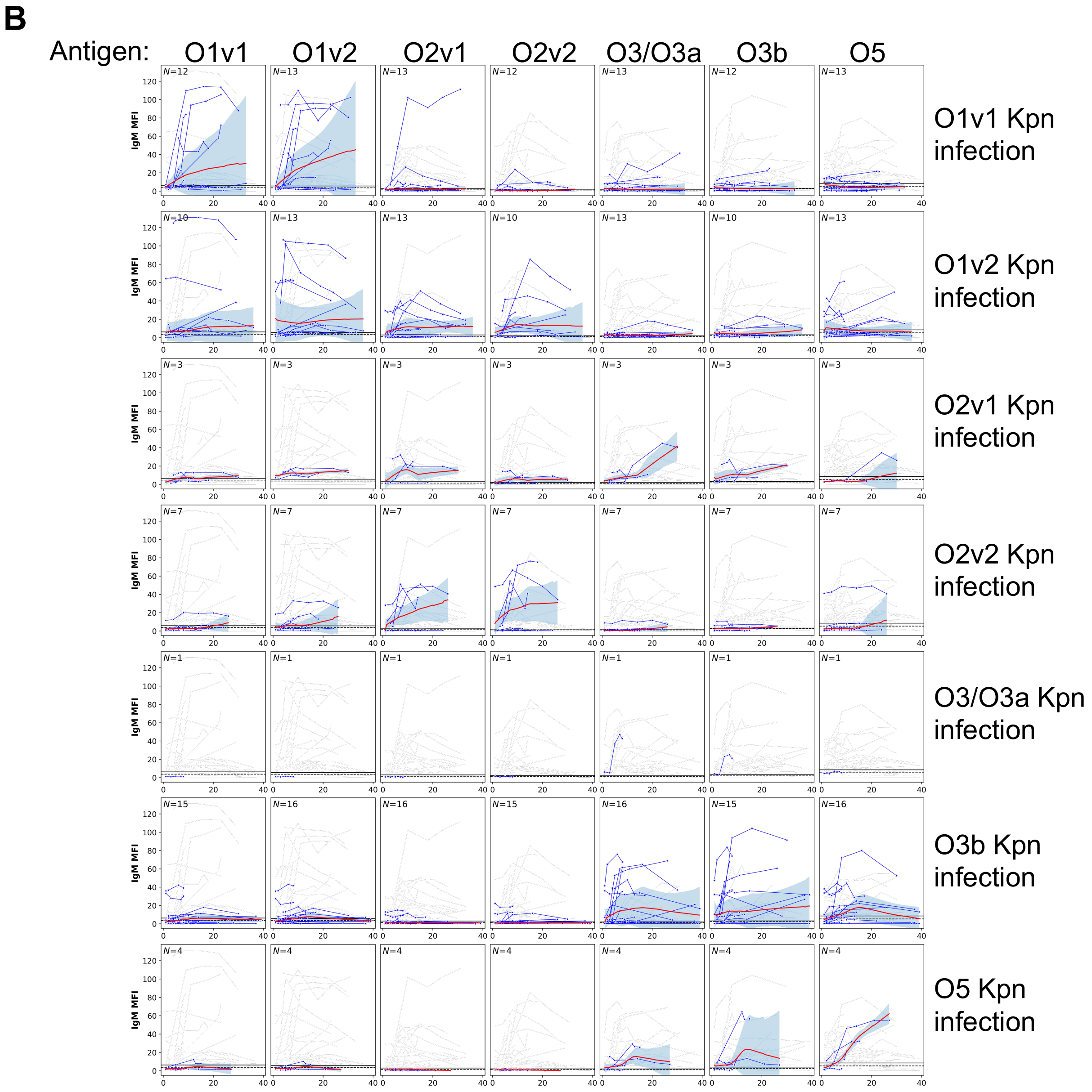
B
Antigen: O1v1 O1v2 O2v1 O2v2 O3/O3a O3b O5
IgM MFI
O1v1 Kpn infection
O1v2 Kpn infection
O2v1 Kpn infection
O2v2 Kpn infection
O3/O3a Kpn infection
O3b Kpn infection
O5 Kpn infection

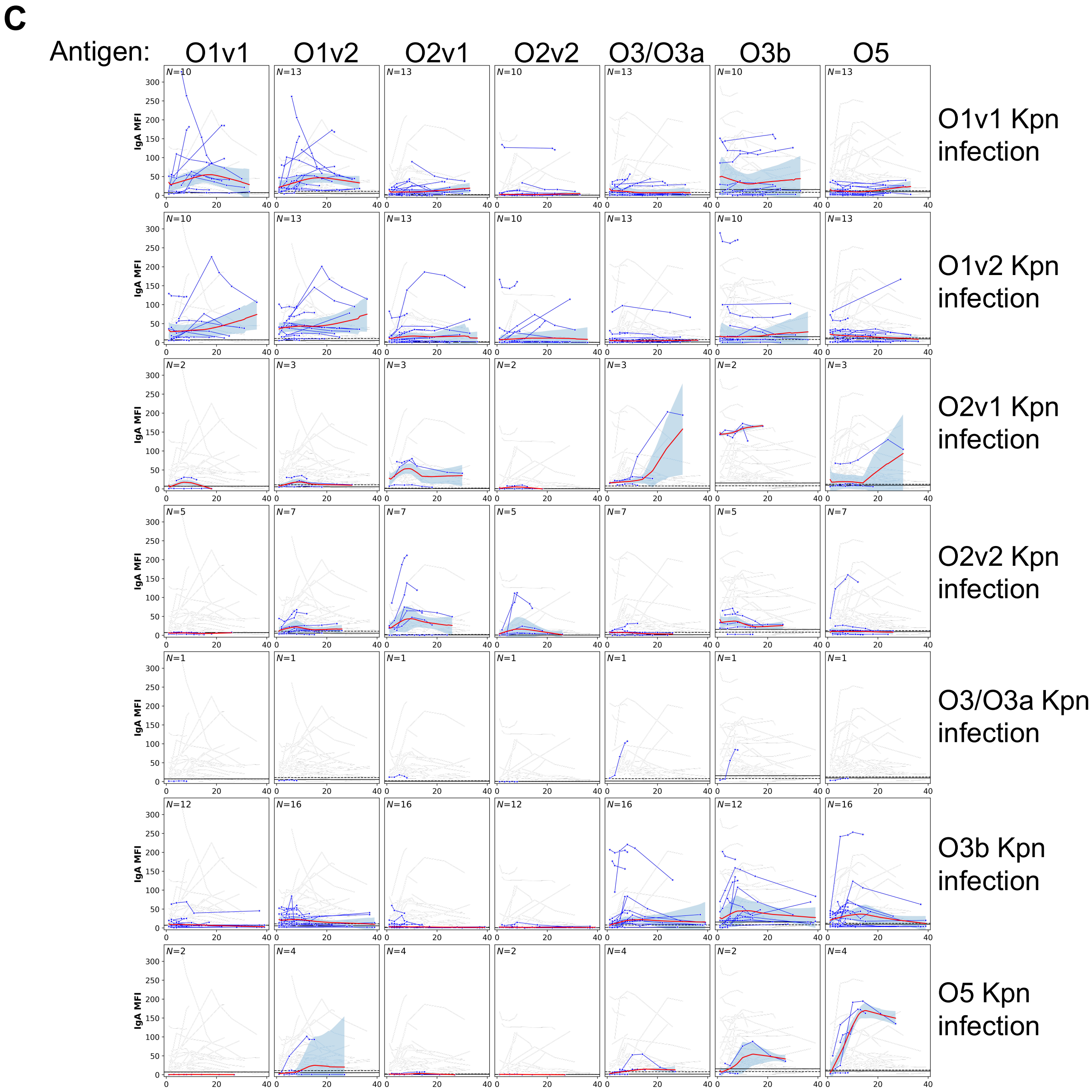

This figure presents the longitudinal IgG **(A)**, IgM **(B)**, and IgA **(C)** antibody responses to O1v1, O1v2, O2v1, O2v2, O3/O3a, O3b, and O5 antigens in patients infected with the corresponding Kpn strains. The antibody responses from patients infected with O1v1, O1v2, O2v1, O2v2, O3/O3a, O3b, and O5 Kpn strains are illustrated as blue lines in their respective rows. The Y-axis represents the Median Fluorescence Intensity (MFI), divided by 1000, while the X-axis denotes the date of plasma collection from patients with Kpn BSI, starting from the first positive blood culture for Kpn (Day 0). Red lines indicate the LOWESS regression applied to the blue lines, with 95% confidence intervals for the LOWESS model shown as shaded blue areas. LOWESS regression analysis was not performed for results with only one case. The solid black line represents the median MFI to antigens from healthy

controls (HC), and the dashed black line represents that from *Enterococcus* spp. bacteremic controls (BC). The number of patients included in each analysis is indicated in each graph.

Figure S9. Antibody responses to OPS and MrkA in patients with Kpn BSI

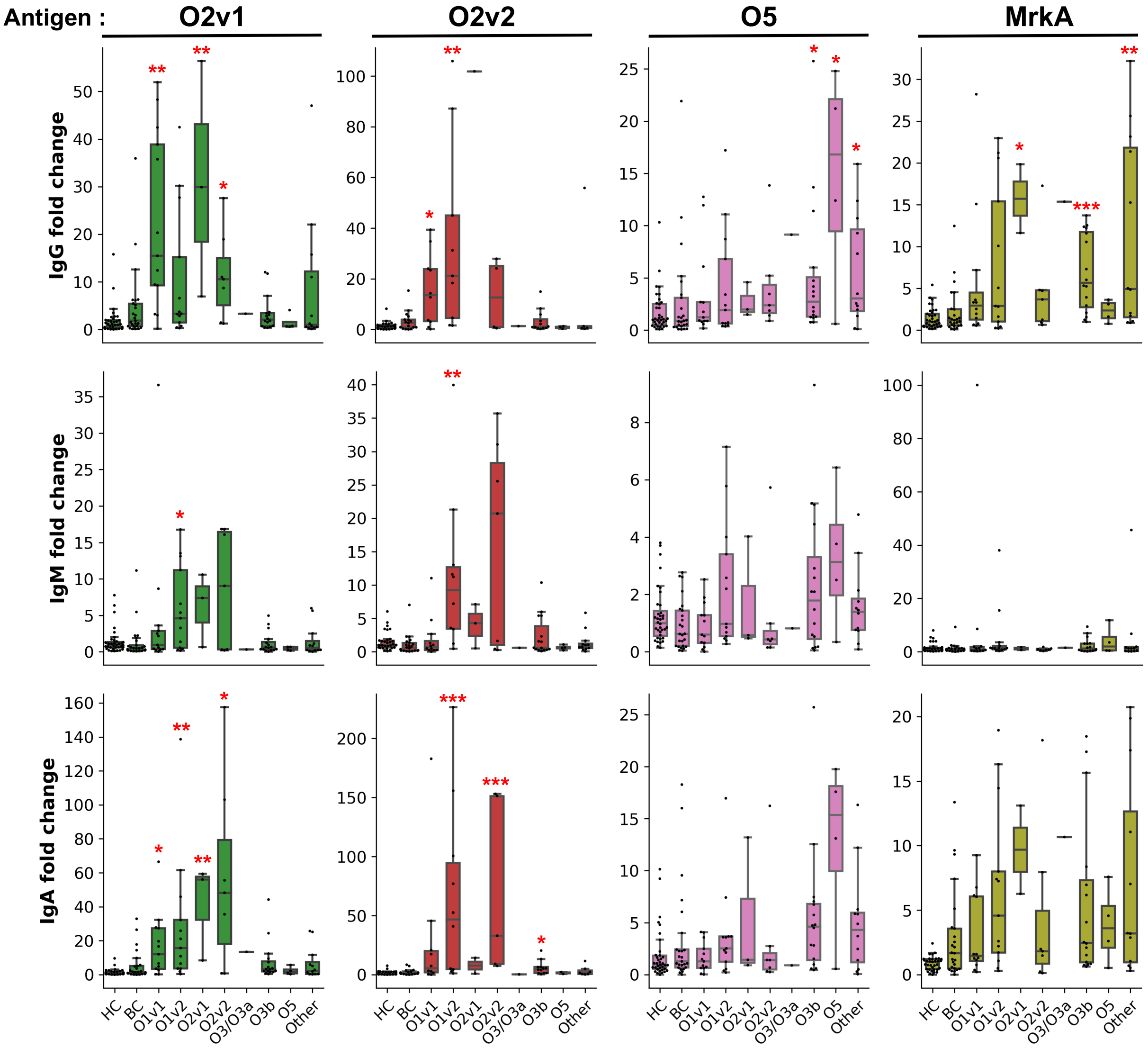

Individual boxplots display IgG, IgM, and IgA antibody responses for healthy individuals (HC), bacteremic patients with *Enterococcus* spp. BSI (BC), and the highest antibody responses from patients with Kpn BSI in response to O2v1, O2v2, O5, and MrkA antigens. The X-axis categorizes plasma samples sourced from HC, BC, and patients infected with Kpn with various OPS types. The "Other" category includes OPS types O4, O12, and unidentified OPS. The Y-axis represents the fold change in antibody responses relative to HC, calculated as the MFI divided by the median MFI of HC. Asterisks indicate the higher P values as determined by Mann-Whitney U test, comparing Kpn BSI to both HC and BC. Significance levels are denoted as follows: * for $0.01 \leq P < 0.05$, ** for $0.001 \leq P < 0.01$, and *** for $P < 0.001$. Detailed information regarding the number of samples is available in Table S2.

Figure S10. OPS-specific ADNP and ADCD responses.

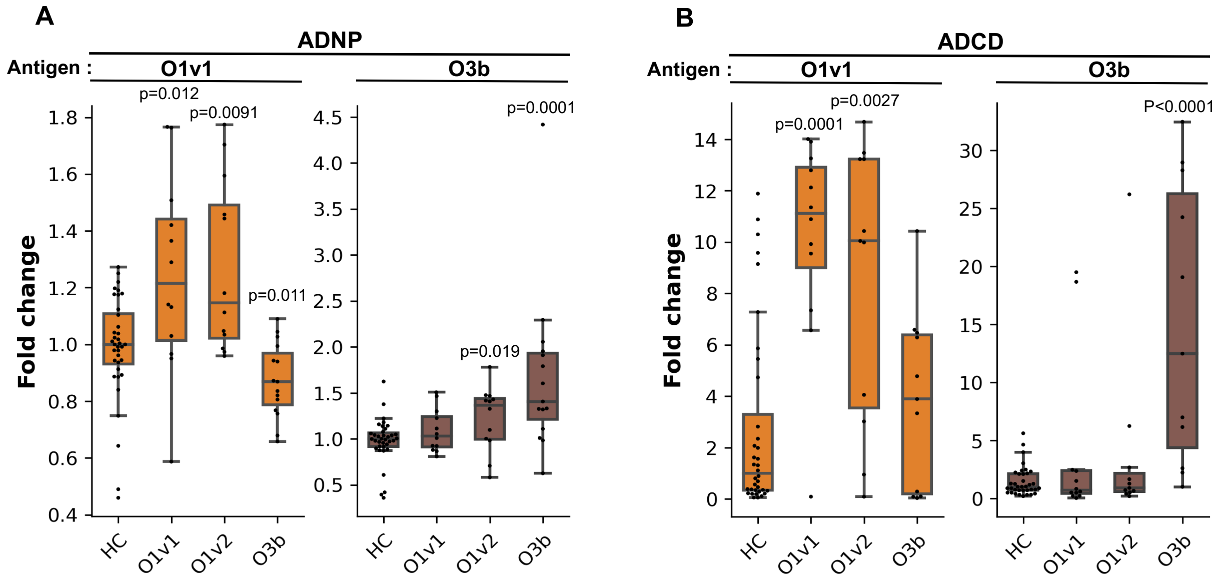

(A) ADNP (Antibody Dependent Neutrophil Phagocytosis) was tested using plasma samples from healthy individuals (HC) and patients with O1v1, O1v2, and O3b Kpn BSI. The ADNP activity of O1v1 and O3b-conjugated beads was normalized to the median ADNP activity in HC. The average fold change of ADNP activities, derived from two healthy neutrophil donors, is presented (N=36 HC, 12 O1v1, 12 O1v2, 15 O3b) **(B)** ADCD (Antibody Dependent Complement Deposition) assays were conducted using opsonized beads conjugated with O1v1 and O3b antigens, along with plasma from HC and patients with O1v1, O1v2, and O3b Kpn BSI. After opsonization, guinea pig complement was added. ADCD activities were divided by the median ADCD activity in HC, and the results are expressed as fold change (N=36 HC, 12 O1v1, 11 O1v2, 11 O3b). P-values less than 0.05 compared to HC were calculated using the Mann-Whitney U test and are shown.

Figure S11. Capsule quantification of Kpn isolates and capsule knockout mutants

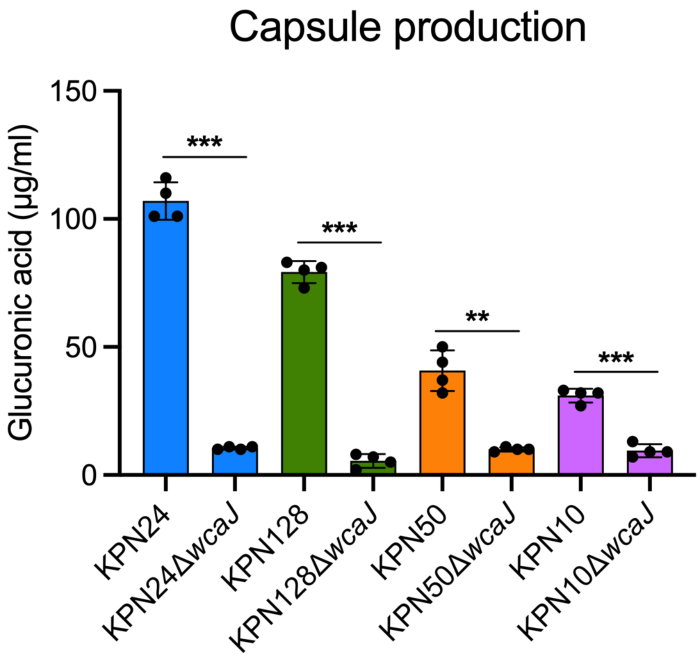

Glucuronic acid levels were quantified using four Kpn wild-type strains (KPN24, KPN128, KPN50 and KPN10) from patients 24, 128, 50 and 10 and their capsule knockout mutants (Δ*wcaJ*) (*N*=4). Asterisks indicate P values smaller than 0.05 by Welch's t-test. Significance levels: * $0.01 \leq P < 0.05$, ** $0.001 \leq P < 0.01$, *** $P < 0.001$.

Figure S12. Flow cytometry gating strategies

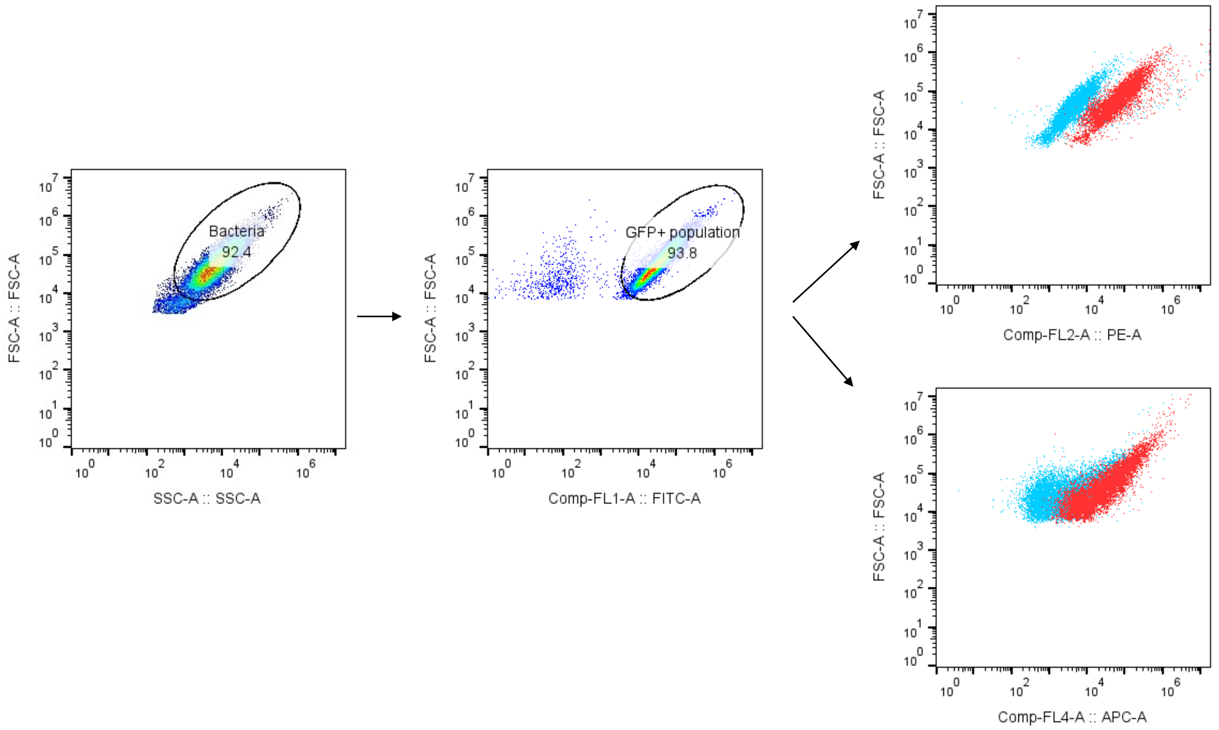

This figure illustrates the flow cytometry gating strategies used in the analysis. The bacterial population was first selected based on SSC-A and FSC-A parameters. Within this gated bacterial population, GFP-positive bacteria were identified using the FL1-A channel. The median PE fluorescence, which indicates IgG binding to bacteria, was measured using the FL2-A channel. Additionally, the median Alexa Fluor 647 fluorescence, representing the binding of anti-complement C3 polyclonal IgG, was measured using the FL4-A channel.

Table S1. Kleborate results of 69 *Klebsiella pneumoniae* isolates

Kpn	Isolation date	K type (CPS)	O type (OPS) **	*rmpADC*	*rmpA2*	Aerobactin	Salmochelin
KPN1	7/24/2021	K14	O3b	-	-	-	-
KPN2	7/30/2021	KL110	O3b	-	-	-	-
KPN3	8/24/2021	K3	O1v2	-	-	-	-
KPN4	9/6/2021	K38	O12	-	-	-	-
KPN5	9/12/2021	K14	O3b	-	-	-	-
KPN6	9/9/2021	K31	O2v1	-	-	-	-
KPN7	9/14/2021	K81	O1v2	-	-	-	-
KPN8	9/18/2021	K5	O3b	-	-	-	-
KPN9	9/18/2021	K52	unknown (OL103)	-	-	-	-
KPN10	9/17/2021	K19	O1v2	-	-	-	-
KPN11	9/20/2021	K15	O4	-	-	-	-
KPN14	9/25/2021	K51	O12	-	-	-	-
KPN15	9/28/2021	K56	unknown (OL103)	-	-	-	-
KPN17	9/27/2021	KL114	O1v1	-	-	-	-
KPN18	9/29/2021	K9	O2v2	-	-	-	-
KPN23	10/4/2021	KL169	unknown (OL104)	-	-	-	-
KPN24	10/2/2021	K2	O1v1	*rmp* 3; ICEKp1	-	-	iro 3 (truncated)
KPN25	10/6/2021	K28	O1v2	-	-	-	-
KPN27	10/18/2021	K21	O3b	-	-	-	-
KPN28	10/17/2021	KL128	O3b	-	-	-	-
KPN31	10/24/2021	K2	O2v1 -> O1v1	-	-	-	-
KPN32	10/27/2021	KL137	unknown (OL101)	-	-	-	-
KPN33	10/28/2021	KL127	unknown (OL101)	-	-	-	-
KPN35	10/29/2021	K2	O1v1	*rmp* 1; KpVP-1	*rmpA2_9*-55%*	iuc 1	iro 1
KPN36	10/28/2021	K46	O3b	-	-	-	-
KPN38	11/7/2021	KL103	O1v1	-	-	-	-
KPN39	11/7/2021	KL110	O3b	-	-	-	-
KPN40	11/8/2021	KL113	O1v2	-	-	-	-
KPN43	11/13/2021	K62	O1v2	-	-	-	-
KPN45	11/11/2021	KL174	O1v2	-	-	-	-
KPN50	11/26/2021	K18	O1v1	-	-	-	-
KPN52	12/1/2021	K21	O3b	-	-	-	-
KPN53	12/3/2021	K46	O3b	-	-	-	-
KPN55	12/6/2021	K25	O1v1	-	-	-	-
KPN57	12/12/2021	KL174	O1v2	-	-	-	-

KPN58	12/13/2021	KL102	O2v2	-	-	-	-
KPN59	12/16/2021	KL108	O1v2	-	-	-	-
KPN63	12/31/2021	K39	O3/O3a	-	-	-	-
KPN66	1/6/2022	KL102	O2v2	-	-	-	-
KPN68	1/16/2022	KL122	O2v2	-	-	-	-
KPN72	1/19/2022	K25	O5	-	-	-	-
KPN73	1/19/2022	K45	O1v2	-	-	-	-
KPN74	1/16/2022	KL117	O1v2	-	-	-	-
KPN75	1/24/2022	K14	O3b	*rmp* 1; KpVP-1 (truncated)	*rmpA2_5*-54%	iuc 1	-
KPN80	2/12/2022	KL102	O2v2	-	-	-	-
KPN81	2/14/2022	KL145	O4	-	-	-	-
KPN87	3/4/2022	KL123	O5	-	-	-	-
KPN89	3/17/2022	K13	O3b	-	-	-	-
KPN90	3/26/2022	KL116	O2v1	-	-	-	-
KPN91	3/26/2022	K20	O2v1	-	-	-	-
KPN92	3/26/2022	K13	O2v1 -> O1v1	-	-	-	-
KPN93	3/27/2022	K11	O5	-	-	-	-
KPN98	5/3/2022	KL102	O3b	-	-	-	-
KPN100	5/4/2022	K38	O1v1	-	-	-	-
KPN102	5/7/2022	K17	O1v1	-	-	-	-
KPN104	5/25/2022	K25	O5	-	-	-	-
KPN105	5/26/2022	K16	O1v1	-	-	-	-
KPN106	5/29/2022	K1	O1v2	-	-	-	-
KPN109	6/12/2022	K52	unknown (OL101)	-	-	-	-
KPN116	7/4/2022	KL102	O2v2	-	-	-	-
KPN118	7/6/2022	KL155	unknown (OL101)	-	-	-	-
KPN120	7/8/2022	KL151	O4	-	-	-	-
KPN121	7/9/2022	KL112	O1v2	-	-	-	-
KPN123	7/12/2022	KL102	O2v2	-	-	-	-
KPN124	7/21/2022	KL125	O3b	-	-	-	-
KPN126	7/23/2022	K49	O1v1	-	-	-	-
KPN127	7/25/2022	KL116	O1v1	-	-	-	-
KPN128	7/31/2022	K57	O3b	*rmp 1*; KpVP-1	*rmpA2_8**	iuc 1	iro 1
KPN129	8/4/2022	K7	O3b	*rmp 1*; KpVP-1	*rmpA2_8**	iuc 1	iro 1

The table presents data on *Klebsiella pneumoniae* (Kpn) isolates, including their isolation dates, capsular polysaccharide (CPS) and O-specific polysaccharide (OPS) types, as well as the presence or absence of the *rmpA*, *rmpA2*, aerobactin, and salmochelin genes as identified through Kleborate analysis. Isolates classified as hypervirulent Kpn (hvKp), containing either a complete *rmpA* or *rmpA2* gene along with complete aerobactin and salmochelin genes, are highlighted in the table with orange boxes.

** In the case of KPN31 and KPN92, the *wbbY* gene was found across different contigs in the BlastN analysis. Consequently, the O2v1 antigen serotypes of these strains were reclassified as O1v1.

Table S2. The number of plasma samples used in Figure 3, Figure 4B, and Figure S9

IgG	O1v1	O1v2	O2v1	O2v2	O3/O3a	O3b	O5	MrkA
**HC**	31	36	36	31	36	31	36	36
**BC**	17	25	25	17	25	17	25	25
**O1v1**	10	13	13	10	13	10	13	12
**O1v2**	9	13	13	9	13	9	13	13
**O2v1**	1	3	3	1	3	1	3	2
**O2v2**	4	7	7	4	7	4	7	7
**O3/O3a**	1	1	1	1	1	1	1	1
**O3b**	12	16	16	12	16	12	16	16
**O5**	3	4	4	3	4	3	4	4
**other**	5	12	12	5	12	5	12	12

IgM	O1v1	O1v2	O2v1	O2v2	O3/O3a	O3b	O5	MrkA
**HC**	33	36	36	33	36	33	36	36
**BC**	22	25	25	22	25	22	25	25
**O1v1**	12	13	13	12	13	12	13	12
**O1v2**	10	13	13	10	13	10	13	13
**O2v1**	3	3	3	3	3	3	3	2
**O2v2**	7	7	7	7	7	7	7	7
**O3/O3a**	1	1	1	1	1	1	1	1
**O3b**	15	16	16	15	16	15	16	16
**O5**	4	4	4	4	4	4	4	4
**other**	12	12	12	12	12	12	12	12

IgA	O1v1	O1v2	O2v1	O2v2	O3/O3a	O3b	O5	MrkA
**HC**	33	36	36	33	36	33	36	36
**BC**	18	25	25	18	25	18	25	25
**O1v1**	10	13	13	10	13	10	13	12
**O1v2**	10	13	13	10	13	10	13	13
**O2v1**	2	3	3	2	3	2	3	2
**O2v2**	5	7	7	5	7	5	7	7
**O3/O3a**	1	1	1	1	1	1	1	1
**O3b**	12	16	16	12	16	12	16	16
**O5**	2	4	4	2	4	2	4	4
**other**	7	12	12	7	12	7	12	12

Table S3. Primers used in this study

pKPGFP construction	rpsL_pro_F	attcgttaccaatagctatactgatttcgtcag	For amplifying the *rpsL* promoter sequence of pCasKP-apr
	rpsL_pro_R	gcctttacgcataaatagctcctggttttag	For amplifying the *rpsL* promoter sequence of pCasKP-apr
	sfGFP_F	ccaggagctatttatgcgtaaaggcgaagag	For amplifying the sequence from the start codon of the superfold GFP gene to the *ampR* promoter in pBAD24-sfGFPx1
	sfGFP_R	cgctgatgacatactcttcctttttcaatattattgaag	For amplifying the sequence from the start codon of the superfold GFP gene to the *ampR* promoter in pBAD24-sfGFPx1
	aprR_F	aaaaaggaagagtatgtcatcagcggtggag	For amplifying the *aprR* gene sequence of pCasKP-apr
	aprR_R	cttggtctgacagtcagccaatcgactggcg	For amplifying the *aprR* gene sequence of pCasKP-apr
	pBadGFP_backbone_F	tcgattggctgactgtcagaccaagtttac	For amplifying the sequence from the end of the *ampR* gene to the end of the *araC* gene in pBAD24-sfGFPx1
	pBadGFP_backbone_R	atcagtatagctattggtaacgaatcagacaattg	For amplifying the sequence from the end of the *ampR* gene to the end of the *araC* gene in pBAD24-sfGFPx1
pDMS197-apr contruction	pDMS197_backbone_F	aataaacaaaacatgagaattgatcctttttgtccggtgttg	For amplifying the plasmid sequence without *tcR* and *tet* promoter sequences in pDMS197
	pDMS197_backbone_R	gattggctgaatggaagccggcggcacc	For amplifying the plasmid sequence without *tcR* and *tet* promoter sequences in pDMS197
	ampRpro_aprR_F	cggcttccattcagccaatcgactggcg	For amplifying the sequence of the *ampR* promoter and *aprR* gene in pKPGFP
	ampRpro_aprR_R	attctcatgttttgtttatttttctaaatacattcaaatatgtatccgc	For amplifying the sequence of the *ampR* promoter and *aprR* gene in pKPGFP
Primers for confirming inserted sequences in plasmids	pSGKP_spacer_F	ctacgggcctaaagaactaa	For amplifying the sequence near the spacer to confirm the spacer sequence in the pSGKP-spec plasmid
	pSGKP_spacer_R	tcacacaggaaacagctatg	For amplifying the sequence near the spacer to confirm the spacer sequence in the pSGKP-spec plasmid
	pDMS197-apr_F	atcaatgattttctggtgcg	For amplifying the sequence near the homologue arm sequences in the pDMS197-apr plasmid
	pDMS197-apr_R	attcaccactccaagaattg	For amplifying the sequence near the homologue arm sequences in the pDMS197-apr plasmid
wcaJ Deletion in KPN10	KPN10_wcaJ_spacer_F	tagtgcttatggagacgagatact	KPN10 *wcaJ* spacer
	KPN10_wcaJ_spacer_R	aaacagtatctcgtctccataagc	KPN10 *wcaJ* spacer
	KPN10_wcaJ_F	ccgatcttgcaattgaggct	For confirming *wcaJ* deletion in KPN10 genome
	KPN10_wcaJ_R	tcaccagtaacagagtcccc	For confirming *wcaJ* deletion in KPN10 genome
	KPN10_up_F	cgatatcgcatgcggtaccttgctagcccaaatgcagg	~0.6 kb upstream sequence of the KPN10 *wcaJ* gene for Gibson assembly with pDMS197-apr, digested by XbaI
	KPN10_up_R	tgattgagtgactcccctctctaatcataaaaatg	~0.6 kb upstream sequence of the KPN10 *wcaJ* gene for Gibson assembly with pDMS197-apr, digested by XbaI
	KPN10_down_F	agaggggagtcactcaatcagtagctgatac	~0.6 kb downstream sequence of the KPN10 *wcaJ* gene for Gibson assembly with pDMS197-apr, digested by XbaI
	KPN10_down_R	ccttggatcccaagcttctttgataatcaaagcgaccaag	~0.6 kb downstream sequence of the KPN10 *wcaJ* gene for Gibson assembly with pDMS197-apr, digested by XbaI
	KPN10_HA_F	tgctagcccaaatgcagg	For amplification of the ~1.2 kb homologous arm sequence from pDMS197-apr_KPN10*wcaJ*
	KPN10_HA_R	tgataatcaaagcgaccaagaaa	For amplification of the ~1.2 kb homologous arm sequence from pDMS197-apr_KPN10*wcaJ*
wcaJ Deletion in KPN24	KPN24_wcaJ_spacer_F	tagtgatatccgcgtgactaaagt	KPN24 *wcaJ* spacer
	KPN24_wcaJ_spacer_R	aaacactttagtcacgcggatatc	KPN24 *wcaJ* spacer
	KPN24_wcaJ_F	agaacataagagctacaatatgattttcaa	For confirming *wcaJ* deletion in KPN24 genome
	KPN24_wcaJ_R	ttcggcaatcagctgcat	For confirming *wcaJ* deletion in KPN24 genome
	KPN24_up_F	cgatatcgcatgcggtacctaggatgctaatgtttcgattg	~0.6 kb upstream sequence of the KPN24 *wcaJ* gene for Gibson assembly with pDMS197-apr, digested by XbaI
	KPN24_up_R	gagcatctagaattcaatcactcatttataaacaag	~0.6 kb upstream sequence of the KPN24 *wcaJ* gene for Gibson assembly with pDMS197-apr, digested by XbaI
	KPN24_down_F	tgattgaattctagatgctccttaagacaag	~0.6 kb downstream sequence of the KPN24 *wcaJ* gene for Gibson assembly with pDMS197-apr, digested by XbaI

	KPN24_down_R	ccttggatcccaagcttctttataggtcacacacggctc	~0.6 kb downstream sequence of the KPN24 *wcaJ* gene for Gibson assembly with pDMS197-apr, digested by XbaI
	KPN24_HA_F	aggatgctaatgtttcgattg	For amplification of the ~1.2 kb homologous arm sequence from pDMS197-apr_KPN24*wcaJ*
	KPN24_HA_R	tataggtcacacacggctc	For amplification of the ~1.2 kb homologous arm sequence from pDMS197-apr_KPN24*wcaJ*
wcaJ Deletion in KPN50	KPN50_wcaJ_spacer_F	tagtgtgcctaaagtttcgatcca	KPN50 *wcaJ* spacer
	KPN50_wcaJ_spacer_R	aaactggatcgaaactttaggcac	KPN50 *wcaJ* spacer
	KPN50_wcaJ_F	tgatagtcgatacgcctcct	For confirming *wcaJ* deletion in KPN50 genome
	KPN50_wcaJ_R	tgggctatagggtttgcatc	For confirming *wcaJ* deletion in KPN50 genome
	KPN50_wcaJ_dsDNA	acatcaattactgtgattaataaaatatataagtaagagggatatctttttttaaaaatctcacagtgagttttcaacgaaatgaaatgt	90 bp of *wcaJ* upstream 45 bp and *wcaJ* downstream 45 bp of KPN50
wcaJ Deletion in KPN128	KPN128_up_F	cgatatcgcatgcggtacctgagctagtcctaatgcgg	~0.6 kb upstream sequence of the KPN128 *wcaJ* gene for Gibson assembly with pDMS197-apr, digested by XbaI
	KPN128_up_R	gcataatgaaagttacacgcctacttatg	~0.6 kb upstream sequence of the KPN128 *wcaJ* gene for Gibson assembly with pDMS197-apr, digested by XbaI
	KPN128_down_F	gcgtgtaactttcattatgcttggataaaaacaatg	~0.6 kb downstream sequence of the KPN128 *wcaJ* gene for Gibson assembly with pDMS197-apr, digested by XbaI
	KPN128_down_R	ccttggatcccaagcttcttcccgtgccattgaaaactc	~0.6 kb downstream sequence of the KPN128 *wcaJ* gene for Gibson assembly with pDMS197-apr, digested by XbaI
	KPN128_wcaJ_F	ttgaaaacccagcagatttg	For confirming *wcaJ* deletion in KPN128 genome
	KPN128_wcaJ_R	agcgtatgagttgtctgaaatca	For confirming *wcaJ* deletion in KPN128 genome

Supplementary References

1. Roach DJ, Sridhar S, Oliver E, et al. Clinical and genomic characterization of a cohort of patients with Klebsiella pneumoniae bloodstream infection. *Clin Infect Dis* 2023.
2. Iyer AS, Jones FK, Nodoushani A, et al. Persistence and decay of human antibody responses to the receptor binding domain of SARS-CoV-2 spike protein in COVID-19 patients. *Sci Immunol* 2020; **5**(52).
3. Bolger AM, Lohse M, Usadel B. Trimmomatic: a flexible trimmer for Illumina sequence data. *Bioinformatics* 2014; **30**(15): 2114-20.
4. Wingett SW, Andrews S. FastQ Screen: A tool for multi-genome mapping and quality control. *F1000Res* 2018; **7**: 1338.
5. Bankevich A, Nurk S, Antipov D, et al. SPAdes: a new genome assembly algorithm and its applications to single-cell sequencing. *J Comput Biol* 2012; **19**(5): 455-77.
6. Lam MMC, Wick RR, Watts SC, Cerdeira LT, Wyres KL, Holt KE. A genomic surveillance framework and genotyping tool for Klebsiella pneumoniae and its related species complex. *Nat Commun* 2021; **12**(1): 4188.
7. Russo TA, Marr CM. Hypervirulent Klebsiella pneumoniae. *Clin Microbiol Rev* 2019; **32**(3).
8. Seemann T. Prokka: rapid prokaryotic genome annotation. *Bioinformatics* 2014; **30**(14): 2068-9.
9. Thompson JD, Higgins DG, Gibson TJ. CLUSTAL W: improving the sensitivity of progressive multiple sequence alignment through sequence weighting, position-specific gap penalties and weight matrix choice. *Nucleic Acids Res* 1994; **22**(22): 4673-80.
10. Waterhouse AM, Procter JB, Martin DM, Clamp M, Barton GJ. Jalview Version 2--a multiple sequence alignment editor and analysis workbench. *Bioinformatics* 2009; **25**(9): 1189-91.
11. Rutherford K, Parkhill J, Crook J, et al. Artemis: sequence visualization and annotation. *Bioinformatics* 2000; **16**(10): 944-5.
12. Wantuch PL, Knoot CJ, Robinson LS, et al. A heptavalent O-antigen bioconjugate vaccine exhibits differential functional antibody responses against diverse Klebsiella pneumoniae isolates. *J Infect Dis* 2024.
13. Wang Y, Wang S, Chen W, et al. CRISPR-Cas9 and CRISPR-Assisted Cytidine Deaminase Enable Precise and Efficient Genome Editing in Klebsiella pneumoniae. *Appl Environ Microbiol* 2018; **84**(23).
14. Naito Y, Hino K, Bono H, Ui-Tei K. CRISPRdirect: software for designing CRISPR/Cas guide RNA with reduced off-target sites. *Bioinformatics* 2015; **31**(7): 1120-3.
15. Wantuch PL, Knoot CJ, Robinson LS, et al. Capsular polysaccharide inhibits vaccine-induced O-antigen antibody binding and function across both classical and hypervirulent K2:O1 strains of Klebsiella pneumoniae. *PLoS Pathog* 2023; **19**(5): e1011367.

IRB Protocol Synopses

For Klebsiella patients and Enterococcus controls.

Title: Immune Responses to Klebsiella pneumoniae in Hospitalized Patients
Protocol Number: 2021P001878
Principal Investigator (PI): Jason B. Harris, MD

Purpose
This study aims to evaluate immune responses to Klebsiella pneumoniae (Kpn) infections in hospitalized patients. The study will use excess clinical samples (e.g., blood, stool, and microbiologic isolates) to analyze immune responses and microbiological strain characteristics, focusing on hypervirulent or drug-resistant strains. A comparator cohort of patients with bloodstream infections caused by bacteria other than Kpn (Enterococcus spp.) will also be included.

Study Population
The study will involve current patients at Massachusetts General Hospital (MGH) with bacteremia or Klebsiella pneumoniae infections of interest. Demographic and clinical data, such as age, gender, medical/surgical history, and medications, will be collected.

Study Design and Procedures
- **Human Materials Used:** Excess plasma/serum, whole blood, stool, and microbiologic strains collected during routine clinical care.
- **Source of Materials:** MGH Clinical Laboratories and hospital records.
- **Data Collection Period:** From June 2021 to June 2026.
- **Analysis Methods:** Immune response and microbiological data will be paired with clinical outcomes. Antimicrobial susceptibility and microbiological strain data will also be assessed.
- **Collaborators:** Includes researchers from Washington University in St. Louis (and others)

Data Management
- **Data Storage:** All data will be stored in a secure REDCap database with access restricted to essential study staff.
- **Identifiers:** Limited identifiers (e.g., MRN) will be used to link clinical and laboratory information. Identifiers will be removed upon study completion.
- **Security Measures:** Password-protected and encrypted Mass General Brigham (MGB) computers with antivirus software will be used.

Waiver of Consent
- **Justification for Waiver:** Samples are excess clinical materials that would otherwise be discarded, and the use of identifiers is limited to ensure minimal risk to privacy. The research could not be conducted practicably without a waiver of consent due to the nature of sample collection.
- **Risk Assessment:** Risks are minimal, as samples will be de-identified and securely stored.

Data Sharing and Agreements
- **External Collaborators:**
    - Dr. David Rosen (Washington University School of Medicine in St. Louis, USA)
- **Materials Shared:** Bacterial strains and serum samples (de-identified).
- **Biostatistical Support:** De-identified clinical data shared under a data use agreement.

Ethical Considerations
- **Privacy Protections:** Data will be de-identified before analysis. PHI will be stored in accordance with MGB security standards.
- **Compliance:** The protocol adheres to MGB IRB policies and applicable regulations, including HIPAA.

Attachments

- **Data Collection Form:** Includes variables such as MRN, age, sex, race, ethnicity, medical/surgical history, problem list, medications, and antimicrobial susceptibility data for Klebsiella isolates.

For Health Controls

Title: Measurement of Serological Response to Travel Diseases in International Travelers
Protocol Number: 2019P001392
Principal Investigator (PI): Regina LaRocque

Purpose
This study evaluates the serological response to infectious disease agents (e.g., malaria, arboviruses such as Zika and Dengue) in international travelers. Using venipuncture and finger stick methods, the study aims to determine exposure rates and assess the reliability of finger stick blood sampling for measuring antibody responses pre- and post-travel.

Study Population
Participants will be recruited at the Massachusetts General Hospital (MGH) Travelers' Advice and Immunization Center. The study will include approximately 200 travelers of all ages.

Study Design and Procedures
- **Sample Collection:** Blood samples will be collected via venipuncture and finger stick before travel (Visit #1) and 14 days post-travel (Visit #2, +/- 7 days).
- **Volume Collected:**
  - Adults: Up to 20 ml venous blood
  - Ages 5–17: 3–5 ml
  - Under 5 years: 1–3 ml
- **Storage and Analysis:** Serum samples and dried blood spots will be stored at -80°C for analysis, including ELISA and pathogen detection (e.g., PCR).
- **Questionnaires:** Participants will complete a post-travel questionnaire to document travel details, symptoms, and medication use.

Specific Aims
1. Assess the frequency of exposure to infectious agents during international travel.
2. Correlate serological results between venipuncture and finger stick methods.
3. Determine the efficiency and reliability of finger stick sampling for measuring immune responses to infectious diseases.

Enrollment and Consent
- **Method of Enrollment:** Travelers at the MGH Travel Clinic will be approached by study staff.
- **Consent Process:** Written informed consent will be obtained, with assent required for children aged 7–17. For non-English speakers, interpreters will assist in the consent process following institutional guidelines.

Data Management
Data will be stored securely in the REDCap database. De-identified samples will be coded with a research ID, and access to identifiers will be limited to essential study staff.

Biostatistical Analyses
Data collected will be analyzed using SAS software. The study will assess antibody prevalence and immune response patterns using descriptive and inferential statistics.

Risks and Discomforts
- Minimal risks include localized pain, redness, or swelling at puncture sites and potential loss of confidentiality.
- Participants will be informed of these risks and advised to seek medical care for symptoms experienced during or after travel.

Potential Benefits

There is no direct benefit to participants. The study will enhance understanding of asymptomatic presentations of diseases such as malaria, Zika, and Dengue among travelers.

Monitoring and Quality Assurance

Data will be managed and monitored by the MGH Biostatistics Center. Samples and data will be securely stored and maintained under strict access controls, with appropriate agreements in place for genetic analysis.

Collaborating Institutions

- Samples may be sent to other partner institutions under secure Data Use Agreements for genetic and immunological analysis.
